# Flavor fidelity and variation of freshly stir-fried and commercial ready-to-heat Yuxiang shredded pork using integrated sensory and volatile profiling

**DOI:** 10.3389/fnut.2026.1936328

**Published:** 2026-09-14

**Authors:** Jia Chen, Juping Du, Lili Duan, Liqiang Yan, Yu Fu, Ping Dong, Xuemei Cai, Mingfeng Qiao

**Affiliations:** 1 Sichuan Cuisine Industrialization Engineering Research Center of Sichuan Higher Education Institutions, Sichuan Tourism University, Chengdu, China; 2 Culinary Science Key Laboratory of Sichuan Province, Sichuan Tourism University, Chengdu, China; 3 College of Food Science and Technology, Southwest Minzu University, Chengdu, China; 4 Zhejiang Tourism Vocational College, Hangzhou, China

**Keywords:** flavor fidelity, flavor variation, integrated flavor profiling, ready-to-heat dishes, volatile compound

## Abstract

Flavor fidelity of commercial ready-to-heat Yuxiang shredded pork (YSP) relative to freshly stir-fried YSP remains insufficiently characterized, particularly across products with heterogeneous formulations and processing histories. A standardized freshly stir-fried YSP was therefore used as a reference to benchmark five commercial frozen ready-to-heat products after standardized reheating using sensory evaluation, electronic nose, electronic tongue, free amino acid profiling, headspace-gas chromatography-ion mobility spectrometry (HS-GC-IMS) and headspace-solid-phase microextraction gas chromatography-mass spectrometry (HS-SPME-GC-MS) combined with chemometric and odor activity value (OAV) analyses. HS-GC-IMS and HS-SPME-GC-MS identified 81 and 158 volatile organic compounds, respectively. Orthogonal partial least squares discriminant analysis (OPLS-DA) prioritized 25 discriminatory HS-GC-IMS features, while OAV analysis screened 51 potentially odor-active GC-MS compounds. The freshly stir-fried reference showed stronger terpene- and Allium-related signals, including methanethiol, diallyl sulfide, diallyl disulphide, neral, geraniol, and 2-ethyl-3,5-dimethylpyrazine. In contrast, the pooled commercial group showed higher levels of hexanal, (E)-2-nonenal, (E,E)-2,4-decadienal, eucalyptol, caryophyllene, phenylethyl alcohol, and ethyl benzoate, reflecting greater contributions from oxidation-, heat-, and seasoning-associated volatiles. Cross-platform correlation analysis further revealed coordinated product-level variation among these volatile modules. These findings provide a market-relevant flavor benchmark for screening candidate aroma markers and prioritizing formulation and processing targets to improve flavor fidelity in ready-to-heat Sichuan dishes.

## 1 Introduction

Commercially prepared dishes have become an important segment of the convenience food market because they facilitate rapid meal preparation, standardized catering, and reduced food waste (1). For traditional dishes, however, convenience alone does not ensure acceptance. Their value also depends on retaining the sensory characteristics that define their culinary identity (2). During industrialization, these dishes can be transformed into different commercial product forms, including ready-to-cook, ready-to-eat, and ready-to-heat products, among which ready-to-heat products are especially relevant to represent the final consumption scenario because they are consumed after storage, distribution, and reheating rather than by a single processing step (3). Flavor fidelity, defined as reproduction of the characteristic sensory and chemical profile of the freshly prepared dish, is therefore an important criterion for evaluating industrialized traditional foods.

Research on prepared meat products has often focused on warmed-over flavor (WOF), a characteristic off-flavor associated with reheating (4, 5). Chen et al. (6) reported that WOF development is closely related to lipid- and protein-derived reactions that generate oxidation products and alter aroma retention and release. Previous studies have therefore examined sensory and instrumental detection of WOF (7), identification of key odorants contributing to WOF (8), variation in fatty acid profiles (9), and the evolution of lipid fingerprints during reheating (10). These approaches do not fully describe complex seasoned dishes, where meat-derived aroma coexists with spice-derived volatiles, scallion-ginger-garlic notes, taste-active constituents, and formulation-dependent ingredients. Therefore, flavor divergence should be evaluated as a multidimensional shift in sensory attributes, taste-active compounds, and volatile profiles rather than simply as WOF-related formation.

Yuxiang shredded pork (YSP) is a representative Sichuan dish characterized by coordinated spicy, sour, savory, and sweet attributes (11, 12). Its characteristic flavor is closely associated with short-time, high-intensity stir-frying, continuous agitation, rapid moisture loss, thermal reactions, and immediate release of spice- and scallion-ginger-garlic-derived volatiles (13, 14). However, these transient cooking conditions are difficult to fully reproduce in commercial ready-to-heat YSP products (15). In addition, commercial products from different brands differ in formulation, raw materials, and processing history, which reflects the real market diversity of industrialized prepared dishes but also complicates flavor standardization (16).

The flavor fidelity of commercial ready-to-heat YSP remains insufficiently characterized. It is unclear which reference-like attributes are retained, attenuated, or enriched across current market products. A single analytical dimension cannot capture changes in perceived aroma and taste, taste-active compounds, and volatile chemistry (17). Sensory evaluation therefore measured perceived attributes, while electronic nose (E-nose) and electronic tongue (E-tongue) analyses provided rapid instrumental fingerprints. Free amino acid (FAA) profiling characterized taste-active components. Headspace solid-phase microextraction gas chromatography-mass spectrometry (HS-SPME-GC-MS) provided tentative molecular annotations and odor activity value (OAV)-based prioritization. Headspace gas chromatography-ion mobility spectrometry (HS-GC-IMS) supplied a complementary volatile fingerprint (18, 19). Together, these methods linked sensory perception, taste-related chemistry, volatile fingerprints, and molecular odorant information, thereby providing a more comprehensive evaluation of complex flavor variation in commercial ready-to-heat YSP.

In this study, a standardized freshly stir-fried YSP was used as a traditional flavor reference, and five commercial frozen ready-to-heat YSP products were evaluated after standardized reheating. Sensory evaluation, E-nose, E-tongue, FAAs, HS-SPME-GC-MS, and HS-GC-IMS were combined to compare sensory, taste-related, and volatile profiles. Chemometric analysis was used to select discriminatory HS-GC-IMS features, odor activity value (OAV) was used to prioritize potentially odor-active GC-MS compounds, and cross-platform correlation analysis was applied to examine associations among representative volatile markers. This integrated sensory and chemical profiling strategy was designed to identify shared flavor features and candidate chemical markers showing consistent differences between the freshly stir-fried reference and commercial products, thereby supporting flavor benchmarking and quality optimization of ready-to-heat traditional dishes.

## 2 Materials and methods

### 2.1 Sample preparation

A freshly stir-fried YSP prepared according to DB51/T1728-2014 served as the standardized reference (CK). Five commercial frozen ready-to-heat YSP products from different brands served as market samples (YSP1-YSP5). CK-1 to CK-3 denote replicate analytical portions of the standardized CK preparation and not independent cooking batches. For each commercial brand, three packages from the same production batch and shelf-life period were purchased and analyzed separately. These packages were treated as package-level analytical units, not independent manufacturing batches. Consequently, the design supports comparison of the tested products but not population-level inference across production batches. Supplementary Table S1 summarizes product form, labeled ingredients, storage conditions, and reheating instructions.

All commercial products were reheated using the same induction cooker and non-stick pan. Reheating continued until the core temperature reached 75 °C and was maintained for 1 min, as measured with a digital probe thermometer. Samples were mixed immediately after reheating. One portion was used for sensory evaluation within 30 min, and the remaining portion was homogenized for instrumental analyses. Unless otherwise stated, instrumental analyses were completed within 24 h after storage at 4 °C.

### 2.2 Sensory evaluation

Sensory evaluation was conducted following Liang et al. (20), with the scoring criteria provided in Supplementary Table S2. Ten trained panelists (5 males and 5 females, 23–25 years old), all postgraduate students in food science, evaluated CK and the five commercial products. Each 50.0 ± 0.5 g sample was presented on plates, with a randomized order for each replication. All evaluations were conducted in individual sensory booths under controlled conditions (22 ± 1 °C and 50 ± 5% relative humidity). Panelists rinsed their mouths with water between samples, and all samples were served under identical conditions. The exercise was a structured trained scoring assay rather than formal quantitative descriptive analysis. A separate model of panelist repeatability, discriminability, and consensus was not fitted. Sensory results are therefore interpreted as comparative screening measurements and are reported as the mean score for each attribute and sample.

Written informed consent was obtained from all assessors before the sensory evaluation. The study involved only non-invasive sensory assessment of edible food samples by informed adult participants. Therefore, according to the applicable institutional requirements, formal ethical approval was not required.

### 2.3 Analysis of taste-related properties

#### 2.3.1 Analysis of E-tongue

The taste characteristics were evaluated using an α-ASTREE E-tongue (Alpha M.O.S., Toulouse, France), with seven ion-selective sensors, including AHS (sourness), CTS (saltiness), NMS (umami), and four cross-sensitive electrodes (PKS, ANS, SCS, CPS). Each 10.0 g sample was diluted with deionized water to 100 ml and ultrasonically extracted for 35 min. The filtrate was measured in triplicate, and the mean of the final five stable readings was used. Data were acquired for 120 s at 1 s intervals with stirring at 1 r/s.

#### 2.3.2 Analysis of free amino acids (FAAs)

FAAs were determined using a published method with minor modifications (21). Triplicate 10.0 g samples of each type were homogenized with 50 ml of 9 % (w/v) sulfosalicylic acid for 3 min using a homogenizer (SCIENTZ 150A, Ningbo Scientz Biotechnology Co., Ltd., Ningbo, Zhejiang, China). The homogenates were subjected to ultrasonic extraction at 30 °C for 40 min to facilitate protein precipitation and release of FAAs. The pH of the mixture was adjusted to 2.2 using HCl and NaOH, followed by centrifugation at 10,000 × g for 10 min, and the resulting supernatants were filtered through 0.22 μm membranes to remove residual particulates. FAAs were analyzed using an amino acid analyzer (S433D, SYKAM, Munich, Germany) with an LCA K07/Li column (150 mm × 4.6 mm) under the following conditions: dual-wavelength detection at 570.0 nm and 440.0 nm, reactor temperature of 130 °C, column temperature from 38 °C to 74 °C, injection volume of 50 μl, total runtime of 108 min, and ninhydrin reagent flow rate of 0.25 ml/min.

### 2.4 Analysis of odor-related properties and volatile compounds

#### 2.4.1 Analysis of E-nose

The overall odor characteristics were analyzed using the Fox 4,000 E-nose system (Alpha MOS, Toulouse, France). This system is equipped with 18 metal oxide sensors (Supplementary Table S3), a mass flow controller, and a microcontroller-based acquisition board. The method was adapted from published protocols to improve sensitivity and repeatability. Briefly, 2.0 g of sample was precisely weighed into a 15 ml headspace vial, sealed, and prepared in triplicate. Operating conditions were a 60 s cleaning period, 10 s sensor-zeroing period, 5 s preparation period, 70 s sampling period, and 400 mL/min carrier-gas flow.

#### 2.4.2 Analysis of VOCs by HS-SPME-GC-MS

VOCs were analyzed by HS-SPME-GC-MS using a Trace DSQ GC-MS coupled to a TriPlus autosampler (Thermo Fisher Scientific, Waltham, USA), following Yao et al. (22) with modifications. Homogenized sample (5.0 g) was placed in a 20 ml headspace vial, and 10.0 μl cyclohexanone standard solution (0.48 μg/μl) was added as the internal standard. The sample was equilibrated at 70 °C for 20 min and extracted using a 50/30 μm DVB/CAR/PDMS fiber (Merck KGaA, Darmstadt, Germany) at 70 °C for 30 min. Thermal desorption was carried out in the injector at 250 °C for 5 min in splitless mode. Separation was achieved on a TG-WAXMSB capillary column (30 m × 0.25 mm × 0.25 μm; Thermo Fisher Scientific, Waltham, USA) with helium (>99.99%) at 1.0 mL/min. A multi-stage oven program was used to improve the separation of low- and high-boiling volatiles as follows: 40 °C for 3 min, 3 °C/min to 100 °C, 5 °C/min to 180 °C, and 8 °C/min to 230 °C held for 5 min. The mass spectrometer was operated in EI mode at 70 eV with an ion source temperature of 250 °C and a scan range of m/z 40–500. Compound identification was confirmed by comparing the acquired mass spectra with the National Institute of Standards and Technology (NIST) library, with both Similarity Index (SI) and Reverse Search Index (RSI) required to be greater than 800. Semi-quantitative analysis was performed using the internal standard based on peak area ratios. OAVs were calculated as the ratio of the semi-quantitative content to the reported odor threshold, as shown in Equation 1 (23). Because odor thresholds are matrix-dependent, the calculated OAVs were used as approximate indicators of relative odor potency rather than absolute measures of aroma contribution in the actual food matrix.


$$\begin{array}{l} OAV=\frac{C_{i}}{T_{i}} \end{array}$$ (1)

Formula: Where *C*_i_ represents the VOC content (μg/kg), *T*_i_ refers to the threshold of volatile compounds in water (μg/kg).

#### 2.4.3 Analysis of VOCs by HS-GC-IMS

VOCs were analyzed using HS-GC-IMS with a FlavourSpec system (Gesellschaft für Analytische Sensorsysteme GmbH, Dortmund, Germany), following Chen et al. (21) with modifications to enhance detection sensitivity and reproducibility. The system was equipped with an autosampler and an airtight heated syringe for direct headspace sampling, coupled with a WAX capillary column (30 m × 0.53 mm, RESTEK, USA) for efficient separation. For sample preparation, 3.0 g of homogenized sample was placed in a 20.00 mL headspace vial and equilibrated at 50 °C for 30 min. Subsequently, 500 μl of the headspace gas was injected into the syringe at 85 °C in a non-split mode and analyzed under isothermal conditions at 45 °C. High-purity nitrogen (99.999%) was used as the carrier gas, following a four-stage flow program: 2 ml/min for 2 min, increased to 10 ml/min over 8 min, further to 100 ml/min over 10 min, then to 150 ml/min over 10 min, and maintained at 150 ml/min for 10 min to ensure complete elution. Compound identification was based on retention indices (RI) calibrated with C_4_-C_9_ n-ketones and drift times matched against the NIST 2014 and IMS databases (24). Quantification was based on the integrated peak areas of selected signal peaks, following the method of Guo et al. (18).

### 2.5 Data processing and statistical analysis

The experiment followed a completely randomized design, with at least three package-level analytical replicates for each treatment group. For commercial products, observations corresponded to packages from one production batch; CK observations were analytical portions of one standardized preparation. These observations were not treated as independent manufacturing or cooking batches. Statistical analyses were conducted using SPSS 26 IBM (IBM, Armonk, NY, USA). One-way analysis of variance was applied, followed by *post hoc* comparisons using the LSD test and Duncan's multiple range test, with the significance set at *p* < 0.05. Network visualization was generated using Cytoscape 3.0 employing force-directed algorithms. Multivariate analyses, including principal component analysis (PCA), orthogonal partial least squares discriminant analysis (OPLS-DA) and variable importance in projection (VIP) scores were implemented in SIMCA 14.1 (Umetrics, Sweden). The characteristic fingerprint and difference plots were generated by Gallery Plot and Reporter software (G.A.S., Dortmund, Germany). Further bioinformatics analyses and data visualization were conducted using RStudio (Version1.6.2), Origin 2021 (OriginLab, Northampton, MA, USA) and the web-based tool Chiplot (https://www.chiplot.online/).

## 3 Results and discussion

### 3.1 Sensory properties of freshly stir-fried and commercial ready-to-heat YSP

As shown in Figure 1, clear differences were observed among CK and the commercial products in appearance, characteristic aroma, allium aroma, taste, and texture. CK showed the highest scores for all attributes, whereas YSP4 was the closest to the freshly prepared reference and YSP3 showed the lowest scores across most attributes. The largest differences were observed for characteristic aroma and allium aroma, followed by appearance and taste. The superior sensory performance of CK was consistent with the immediate release and integration of volatile compounds from scallion, ginger, garlic, pickled chili, and other seasonings during stir-frying. By contrast, the lower scores of several commercial ready-to-heat products may be related to cumulative quality changes during pre-processing, frozen storage, distribution, and reheating. Previous studies on prepared meat systems have reported that freezing-thawing-reheating can affect water distribution, moisture release, and microstructural integrity, thereby influencing texture and juiciness (20). In addition, heat-induced changes in muscle proteins and protein-flavor interactions can influence the retention and release of aroma compounds (6). Therefore, the sensory decline observed in the commercial products may be partly explained by processing-related changes in texture, water retention, and aroma release behavior. However, the present market-sampling design cannot isolate these mechanisms from brand-specific formulation effects.

**Figure 1:**
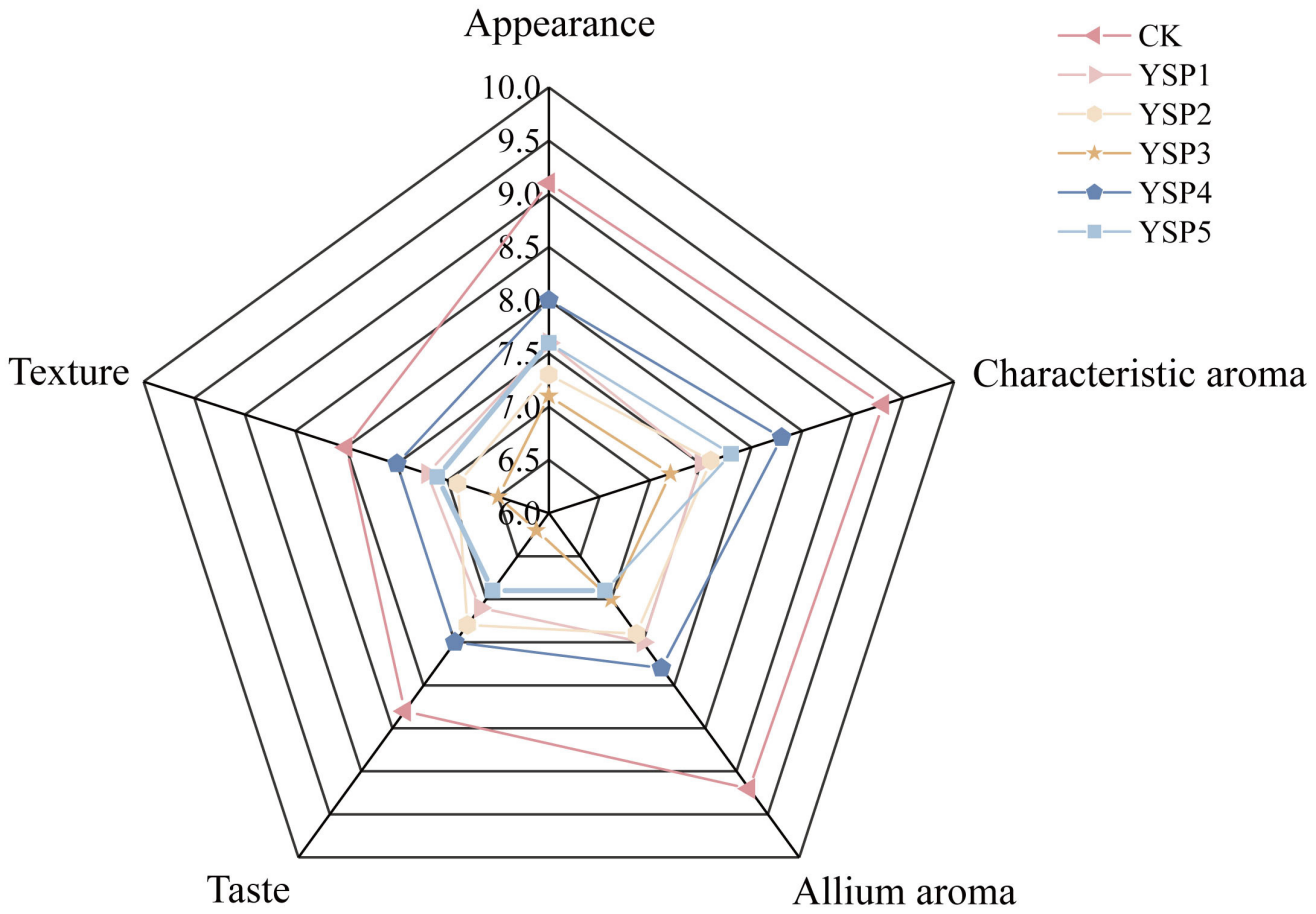
Sensory attribute scores for CK and five commercial ready-to-heat YSP products.

### 3.2 Taste-related properties of freshly stir-fried and commercial ready-to-heat YSP

#### 3.2.1 Analysis of E-tongue

As shown in the radar plot (Figure 2A), the six samples displayed distinct response patterns across the five taste attributes (sourness, saltiness, umami, sweetness, and bitterness). Among these attributes, sweetness and saltiness generally showed stronger response levels than the other dimensions across the samples, whereas umami and sourness exhibited comparatively lower responses. Notably, bitterness displayed the most apparent variation among samples, indicating a higher sensitivity of this attribute for differentiating taste fingerprints within the present dataset.

**Figure 2:**
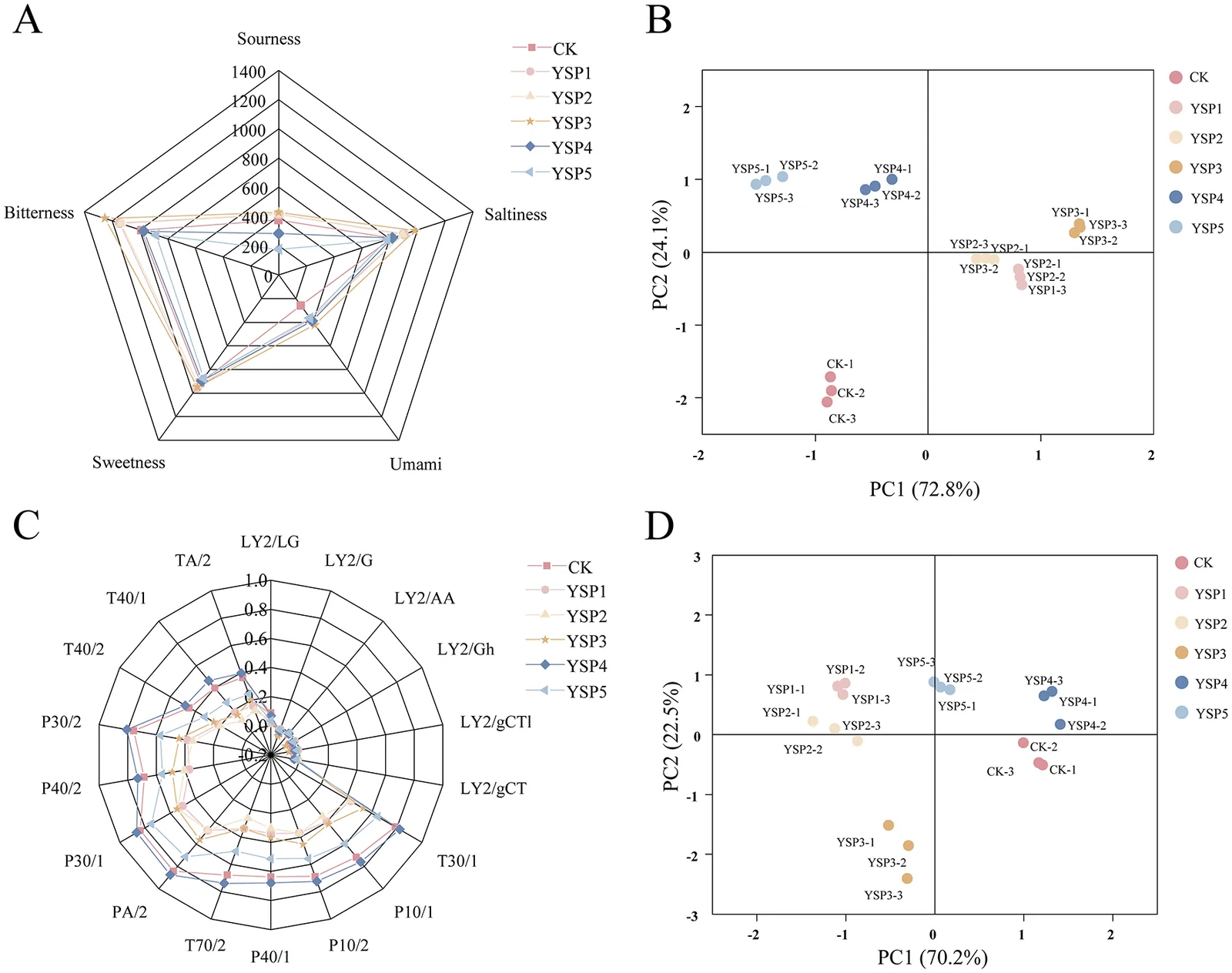
Electronic tongue (E-tongue) and electronic nose (E-nose) responses of CK and five commercial ready-to-heat YSP products. **(A)** E-tongue radar plot; **(B)** E-tongue PCA score plot; **(C)** E-nose radar plot; **(D)** E-nose PCA score plot.

The PCA score plot (Figure 2B) further confirmed the discriminative ability of the E-tongue, with PC1 (72.8%) and PC2 (24.1%) explaining 96.9% of the total variance, thereby capturing the majority of taste-related information. The score plot showed a clear separation between CK and the commercial ready-to-heat samples, indicating distinct taste fingerprints between CK and the commercial products under standardized reheating. Meanwhile, the commercial ready-to-heat samples were not completely overlapped but distributed in distinct regions, implying noticeable inter-brand variability in taste fingerprints. Overall, these results indicate that the E-tongue provides a rapid, reproducible tool for comparing taste-related response patterns between freshly stir-fried and commercial ready-to-heat YSP, and the generated multivariate descriptors can be further integrated with chemical datasets.

#### 3.2.2 Analysis of FAAs

FAAs contribute directly to taste and participate in flavor-forming reactions during heating. A total of 17 FAAs were identified (Table 1). The CK contained 36.84 mg/100 g total FAAs, whereas the commercial products ranged from 21.42 to 39.17 mg/100 g. The umami-related amino acids Asp and Glu showed a modest overall decrease in the commercial ready-to-heat products. This trend is consistent with the reduced retention of water-soluble nitrogenous compounds when upstream precooking and handling weaken water-holding behavior and promote the release of liquid fractions that carry dissolved solutes (24). Arg, a bitter-tasting amino acid, decreased markedly from 4.07 mg/100 g in the freshly stir-fried sample to 0.87–2.05 mg/100 g in the commercial ready-to-heat products. This reduction is consistent with processing-related losses of soluble components and aligns with prior observations that arginine-related treatments can influence water retention and quality in heated meat systems (25). The downward trend of Arg was also directionally consistent with the E-tongue result that bitterness provided higher discrimination among samples, supporting a linkage between bitter-related amino acids and taste fingerprint divergence. Among the five commercial brands, FAA compositions varied substantially, as indicated by wide concentration ranges for Trp (0.10–3.17 mg/100 g), Gly (0.43–3.66 mg/100 g), and His (0.42–0.83 mg/100 g). This pattern indicates a shared difference relative to CK superimposed on substantial brand-specific variability. It also agrees with evidence that freezing, thawing, and reheating can reshape microstructure and water distribution in precooked meat, increasing drip release and thereby altering the retention of soluble components during reheating (26). Overall, FAA profiles differed between CK and the commercial products and also showed pronounced inter-brand variability under standardized reheating.

**Table 1:** Concentration (mg/100 g) and composition of free amino acids obtained from different YSP.

Taste category	FAA	CK	YSP1	YSP2	YSP3	YSP4	YSP5
Umami FAA	Asp	2.39 ± 0.11^a^	1.59 ± 0.08^b^	0.65 ± 0.05^d^	0.83 ± 0.02^c^	0.54 ± 0.05^d^	0.67 ± 0.03^cd^
	Glu	14.13 ± 1.01^a^	8.46 ± 0.42^b^	13.13 ± 1.08^a^	15.35± 1.46^a^	14.04 ±0.97^a^	15.17 ± 1.08^a^
	Gly	1.45 ± 0.11^b^	0.99 ± 0.10^c^	0.84 ± 0.08^cd^	1.24 ± 0.09^bc^	0.43 ± 0.12^d^	3.66 ± 0.33^a^
	Ala	2.27 ± 0.17^b^	2.30 ± 0.21^b^	3.07 ± 0.27^a^	3.09 ± 0.16^a^	1.05 ± 0.10^c^	1.09 ± 0.11^c^
	Lys	1.22 ± 0.12^bc^	1.40 ± 0.02^ab^	1.14 ± 0.03^c^	1.42 ± 0.10^a^	0.47 ± 0.04^d^	0.59 ± 0.05^d^
Sweet FAA	Ser	1.70 ± 0.04^b^	2.01 ± 0.10^a^	1.50 ± 0.08^b^	2.06 ± 0.12^a^	0.61 ± 0.06^c^	0.78 ± 0.06^c^
	Thr	1.45 ± 0.11^a^	1.18 ± 0.11^bc^	0.94 ± 0.06^c^	1.33 ± 0.08^ab^	0.34 ± 0.13^d^	0.46 ± 0.03^d^
	Pro	1.11 ± 0.05^bc^	1.34 ± 0.13^a^	1.04 ± 0.03^c^	1.30 ± 0.09^ab^	0.45 ± 0.03^d^	0.46 ± 0.03^d^
Bitter FAA	Arg	4.07 ± 0.41^a^	1.96 ± 0.19^b^	1.83 ± 0.03^b^	2.05 ± 0.20^b^	0.87 ± 0.07^c^	1.19 ± 0.06^c^
	lle	0.91 ± 0.08^b^	1.09 ± 0.16^b^	0.88 ± 0.02^b^	1.38 ± 0.08^a^	0.36 ± 0.03^c^	0.29 ± 0.02^c^
	Val	1.34 ± 0.05^b^	1.35 ± 0.11^b^	1.19 ± 0.06^b^	1.74 ± 0.08^a^	0.49 ± 0.03^c^	0.47 ± 0.03^c^
	Leu	1.38 ± 0.08^c^	1.78 ± 0.18^b^	1.46 ± 0.12^c^	2.27 ± 0.16^a^	0.60 ± 0.01^d^	0.51 ± 0.03^d^
	Met	0.28 ± 0.03^c^	0.45 ± 0.03^b^	0.44 ± 0.10^b^	0.67 ± 0.06^a^	0.10 ± 0.02^d^	0.22 ± 0.02^cd^
	His	0.27 ± 0.04^e^	0.67 ± 0.02^b^	0.55 ± 0.04^c^	0.83 ± 0.03^a^	0.45 ± 0.04^d^	0.42 ± 0.02^d^
Aromatic amino acids	Tyr	0.72 ± 0.03^b^	0.62 ± 0.04^b^	0.32 ± 0.01^c^	1.11 ± 0.11^a^	0.23 ± 0.06^c^	0.31 ± 0.03^c^
	Phe	0.97 ± 0.03^b^	0.97 ± 0.02^b^	0.72 ± 0.06^c^	1.31 ± 0.10^a^	0.31 ± 0.02^d^	0.31 ± 0.02^d^
	Trp	1.19 ± 0.07^c^	3.17 ± 0.25^a^	1.15 ± 0.02^c^	1.19 ± 0.09^c^	0.10 ± 0.02^d^	2.15v0.15^b^
Total		36.84 ± 1.04^b^	31.31 ± 0.53^c^	30.83 ± 0.52^c^	39.17v1.07^a^	21.42 ± 1.26^e^	28.74 ± 1.35^d^

Values are means ± standard deviations. Different lowercase letters (a–e) within a row indicate significant differences among products (*p* < 0.05).

### 3.3 Odor-related properties and volatile compound characteristics

#### 3.3.1 Analysis of E-nose

The 18 E-nose sensors produced distinct response intensities across the six products (Figure 2C). Across all samples, P30/1, P30/2, PA/2, T30/1 and P10/1 displayed the highest responses, indicating that the headspace profile of YSP was mainly characterized by sensor channels related to polar/oxygenated volatiles (P30/1, P30/2), ethanol/amine-related signals (PA/2), organic-solvent-like volatiles (T30/1), and hydrocarbons (P10/1).

As shown in Figure 2D, CK clustered close to YSP4, whereas YSP1 clustered close to YSP2. PC1 (70.2%) and PC2 (22.5%) together explained 92.7% of the total variance. The other products were more widely separated, confirming substantial inter-brand variation among commercial ready-to-heat products.

#### 3.3.2 Identification of VOCs by HS-GC-IMS and HS-SPME-GC-MS analysis

HS-GC-IMS and HS-SPME-GC-MS revealed both recurrent and brand-specific differences between CK and the commercial products. A total of 81 VOCs (including monomers and dimers) were identified by HS-GC-IMS, including 2 acids, 13 alcohols, 17 aldehydes, 8 esters, 6 furans, 11 ketones, 4 pyrazines, 12 terpenes, 4 sulfides and 4 other compounds (Supplementary Table S4). The HS-GC-IMS two-dimensional spectra (Figure 3A) provided an overview of the volatile profiles of freshly stir-fried and five commercial ready-to-heat products. Most signals were observed within a retention time range of 200.0–1000.0 s and a drift time range of 0.5–1.0 ms. Overall spot density and intensity clearly differed between CK and the commercial ready-to-heat samples, indicating matrix-dependent changes in volatile composition and release behavior. This observation is consistent with the recognized capability of HS-GC-IMS to provide rapid, high-throughput aroma fingerprints for processed foods (27). Differential spectra (Figure 3B) were generated by using CK as the reference spectrum, where red and blue regions indicate higher and lower VOC concentrations relative to CK, respectively. The plots showed clear differences in signal number and intensity among products, indicating substantial variation in VOC abundance.

**Figure 3:**
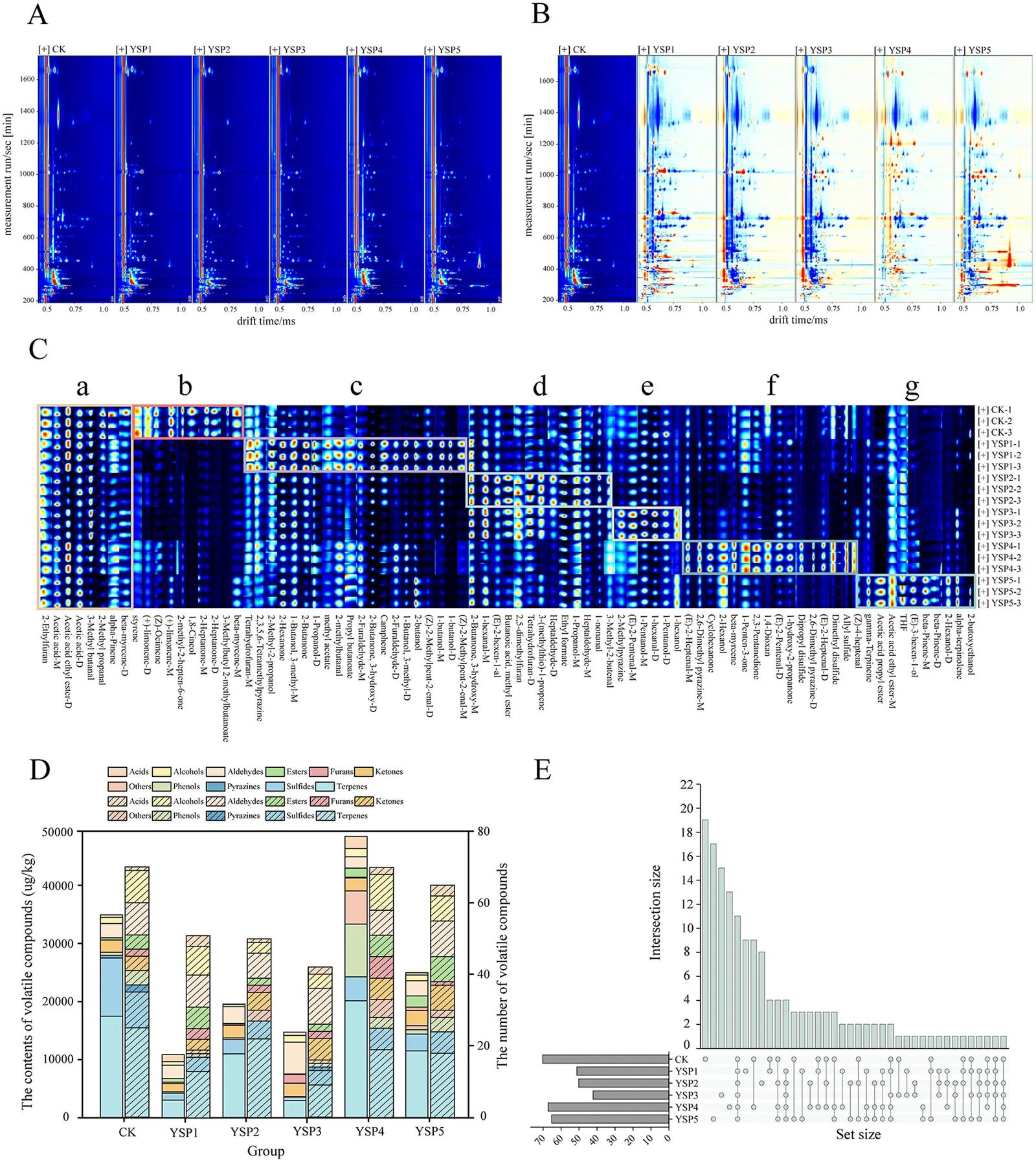
VOC profiles of CK and five commercial ready-to-heat YSP products. **(A)** Two-dimensional HS-GC-IMS topographic plots; **(B)** HS-GC-IMS difference plots using CK as the reference; **(C)** HS-GC-IMS fingerprint map, with rows representing replicates and columns representing volatile signals; **(D)** internal-standard-based semi-quantitative volatile contents (solid fill) and compound counts (hatched fill) by chemical class from HS-SPME-GC-MS; **(E)** UpSet plot of shared and sample-specific GC-MS annotations.

In the IMS fingerprint map (Figure 3C), columns correspond to volatile markers and rows represent sample replicates, with color intensity reflecting relative abundance. Zone a represented the common acid-ester backbone and remained the dominant module across samples. It was mainly driven by acetic acid and ethyl acetate, together with Strecker type aldehydes (2-methyl-propanal and 3-methyl-butanal). Acetic acid is a hallmark volatile of vinegar based seasoning systems and is frequently reported as a key discriminant marker in vinegar aroma profiling (28). Ethyl acetate may form in acid alcohol matrices through the esterification of ethanol and acetic acid, and is frequently detected alongside acetic acid in vinegar-type systems, jointly shaping the characteristic pungent-fruity aroma profile (29). Zone b most clearly distinguished CK from most commercial products. Limonene, (Z)-ocimene, and 1,8-cineole were stronger in CK, indicating a greater contribution from fresh spice-derived top notes. Their lower signals in several commercial products are consistent with volatilization or thermo-oxidative degradation, although formulation differences cannot be excluded (30).

Several commercial products showed stronger carbonyl, furan, ketone, pyrazine, and sulfur signals in Zones c-f. These regions included markers such as 3-hydroxy-2-butanone, 2-butanone, 2-furaldehyde, hexanal, nonanal, heptanal, (E)-2-pentenal, 2,5-dimethylfuran, 2-methylpyrazine, and sulfur-containing volatiles. These compounds are generally associated with thermal reactions, sugar degradation, lipid oxidation, and Allium-derived sulfur chemistry, suggesting that commercial ready-to-heat products tended to show greater contributions from heat- and oxidation-associated aroma signals than the freshly stir-fried reference (31–33). However, these signals were not uniformly enhanced across all commercial samples. Therefore, Zones c-f should be interpreted as regions reflecting both common divergence from the freshly stir-fried reference and inter-brand differences among commercial ready-to-heat products. Specifically, Zone f was most evident in YSP4 and represented a pyrazine- and sulfur-related region. The co-occurrence of pyrazines and sulfur-containing volatiles indicates the combined contribution of thermal reactions and Allium-derived precursors, suggesting a stronger heat-associated aroma signature or a higher contribution of sulfur-containing seasonings in this product (34). Zone g was prominent in YSP5 and contained terpene-, alcohol-, and ester-related markers, indicating that formulation and processing history played an important role in shaping the aroma profile of this brand (35).

HS-SPME-GC-MS tentatively identified 158 VOCs: 41 terpenes, 17 sulfides, 3 pyrazines, 9 phenols, 10 ketones, 5 furans, 18 esters, 17 aldehydes, 23 alcohols, 9 acids, and 6 other compounds (Supplementary Table S5). CK and YSP4 showed relatively high summed semi-quantitative VOC abundances, estimated at 35,069.10 and 51,289.59 μg/kg, respectively, and also contained among the largest numbers of detected VOCs (70 and 67, respectively; Figure 3D). The UpSet plot further summarized the shared and sample-specific GC-MS annotations among CK and the five commercial products (Figure 3E). These results indicate that the commercial products did not show a uniform reduction in VOC abundance relative to CK, but instead exhibited substantial product-specific redistribution of volatile profiles. At the class level, CK was mainly dominated by terpenes (17380.99 μg/kg) and sulfides (10037.41 μg/kg), highlighting the importance of spice-derived front notes and Allium-related pungency in the freshly stir-fried system. Carbonyl-related modules linked to WOF are more pronounced in certain commercial ready-to-heat samples, consistent with an association-based interpretation in which lipid oxidation and protein degradation during production-storage-reheating may contribute to background aroma accumulation (36). Specifically, total aldehydes ranged from 2294.38 μg/kg to 5484.24 μg/kg in the commercial ready-to-heat samples, compared with 2444.75 μg/kg in CK, indicating that some brands showed higher semi-quantitative aldehyde abundances mainly derived from fatty acid oxidation, branched-chain amino acid degradation, and Maillard-related reactions (37). Notably, terpenes and sulfur-containing compounds remained at relatively high levels in the freshly stir-fried sample, which was consistent with the results obtained by HS-GC-IMS.

Together, the platforms identified recurrent and product-specific divergence from CK. HS-GC-IMS resolved fingerprint-level patterns, whereas HS-SPME-GC-MS provided broader tentative annotations and semi-quantitative estimates. These complementary datasets supported exploratory candidate screening and cross-platform comparison.

### 3.4 Differential marker discovery and aroma contribution analysis

#### 3.4.1 Discriminatory volatile features selected from HS-GC-IMS

Different feature-selection strategies were deliberately used because HS-GC-IMS and HS-SPME-GC-MS differ in response scale, separation behavior, and annotation coverage. OPLS-DA was applied to HS-GC-IMS fingerprints to select discriminatory signal features (VIP > 1), whereas GC-MS compounds were prioritized using statistical contrasts and OAVs. In the HS-GC-IMS model, PC1 and PC2 explained 31.9 and 24.9% of the variance, respectively, with a cumulative explanation of 56.8%. The model exhibited excellent fitting and predictive performance (*R*^2^*X* = 0.96, *R*^2^*Y* = 0.98, *Q*^2^ = 0.93; Figure 4A). In 200 permutation tests, the intercepts were *R*^2^ = 0.20 and *Q*^2^ = −0.79 (Figure 4B), supporting model stability within this dataset.

**Figure 4:**
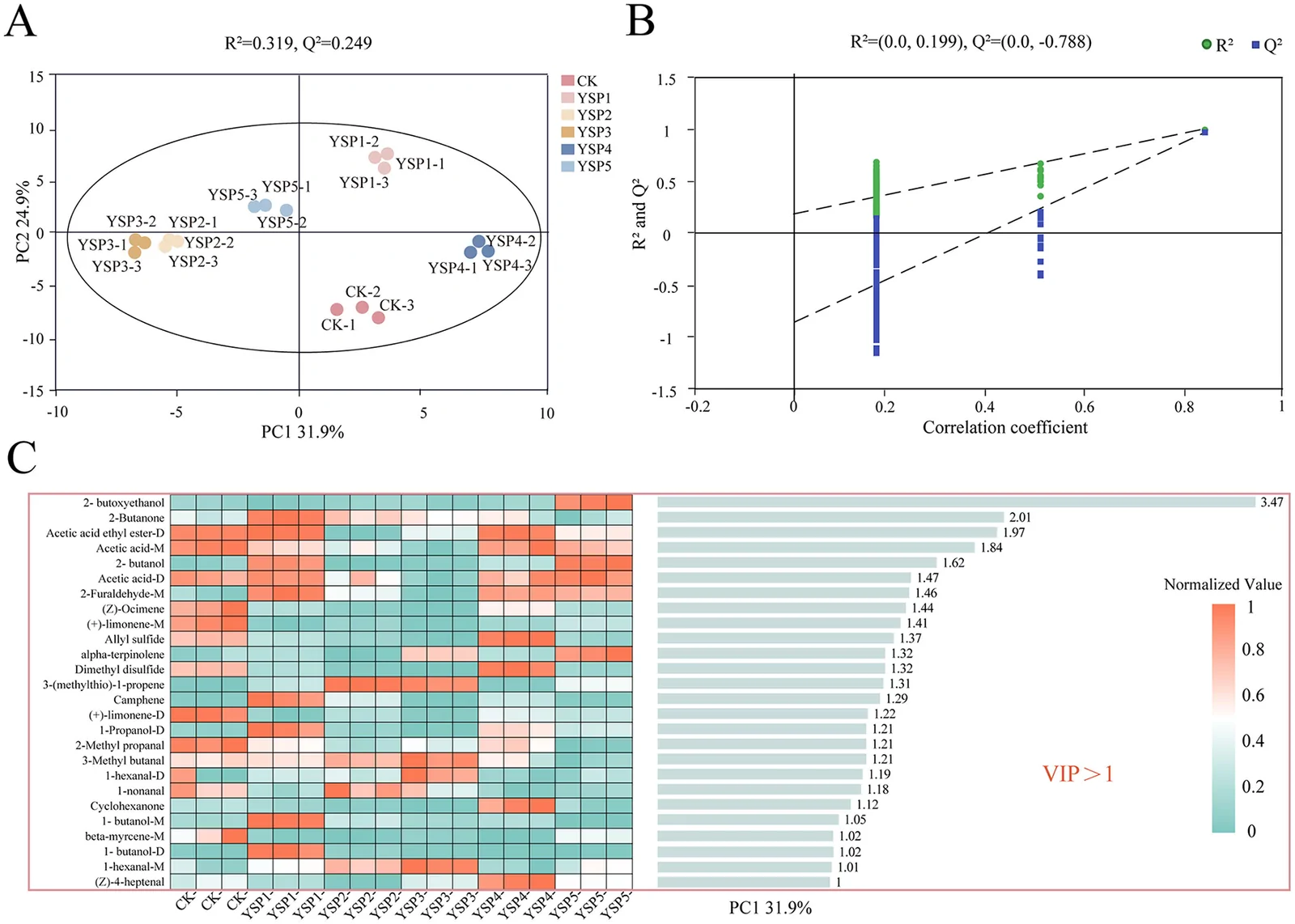
OPLS-DA of HS-GC-IMS volatile profiles in freshly stir-fried and commercial ready-to-heat YSP. **(A)** OPLS-DA score plot; **(B)** 200-permutation validation plot; **(C)** Z-score heat map and VIP values for 25 candidate features (VIP > 1).

The VIP analysis identified 25 HS-GC-IMS features with VIP > 1 (Figure 4C). High-ranking features included acetic acid (M/D), ethyl acetate-D, 3-hydroxy-2-butanone (M/D), 2-butanone, 2-butanol, 3-methyl-1-butanol (M/D), 2-methylpropanal, and 3-methylbutanal. These compounds are associated with fruity, malty, and fatty odor notes (38). By contrast, (Z)-ocimene, (+)-limonene (M/D), camphene, α-terpinolene, 1,8-cineole, allyl sulfide, dimethyl disulfide, and 3-(methylthio)-1-propene showed a significant decrease in commercial products, which are closely associated with the citrus-like, herbal, and garlic-like flavor. Their origin is equally consistent with the formulation and cooking process, since the terpenes are mainly spice-derived, whereas the sulfur volatiles originate from the rapid breakdown and transformation of Allium precursors. In selected brands, 2-furaldehyde and dimethylpyrazine showed stronger signals. These compounds are associated with sugar degradation and Maillard-type chemistry and can contribute roasted or meaty notes (39). Because the design compared unmatched commercial brands, these features are candidate product markers rather than validated markers of reheating.

#### 3.4.2 Potentially odor-active compounds prioritized by HS-SPME-GC-MS

HS-SPME-GC-MS provided a sensory-oriented selection layer. Across the six samples, 51 compounds had OAV > 1 (Supplementary Table S6), comprising 15 terpenes, 11 aldehydes, 5 phenols, 5 sulfides, 5 alcohols, 3 ketones, 4 esters, 1 furan, 1 pyrazine, and 1 other compound (Figure 5A). CK contained 32 compounds with OAV > 1, followed by YSP5 (29), YSP4 (25), YSP2 (20), YSP1 (20), and YSP3 (17). Five compounds occurred with OAV > 1 across all products: 3-methylbutanal, diallyl disulfide, neral, (E)-2-decenal, and 2-pentylfuran. In CK, the highest OAVs were observed for (E,E)-2,4-decadienal (2069.29), methanethiol (907.05), 2-ethyl-3,5-dimethylpyrazine (814.85), nonanal (764.93), geraniol (595.31), and myrcene (378.41), indicating that its aroma profile was jointly shaped by fried-fatty notes, Allium pungency, spice-derived freshness, and roasted nuances (40). At the same time, clear brand-level divergence was observed: YSP2, YSP3, and YSP5 were characterized by very high OAVs of (E,E)-2,4-decadienal (4968.75, 5004.89, and 5640.52, respectively), while YSP3 also showed a particularly high nonanal value (2213.95), and YSP5 was distinguished by (E)-2-nonenal (1958.14). YSP4 exhibited a pronounced phenolic-terpenic character, with eugenol and eucalyptol reaching OAVs of 3606.80 and 2251.56, respectively, suggesting a stronger contribution of clove-like and cineole-rich seasoning notes.

**Figure 5:**
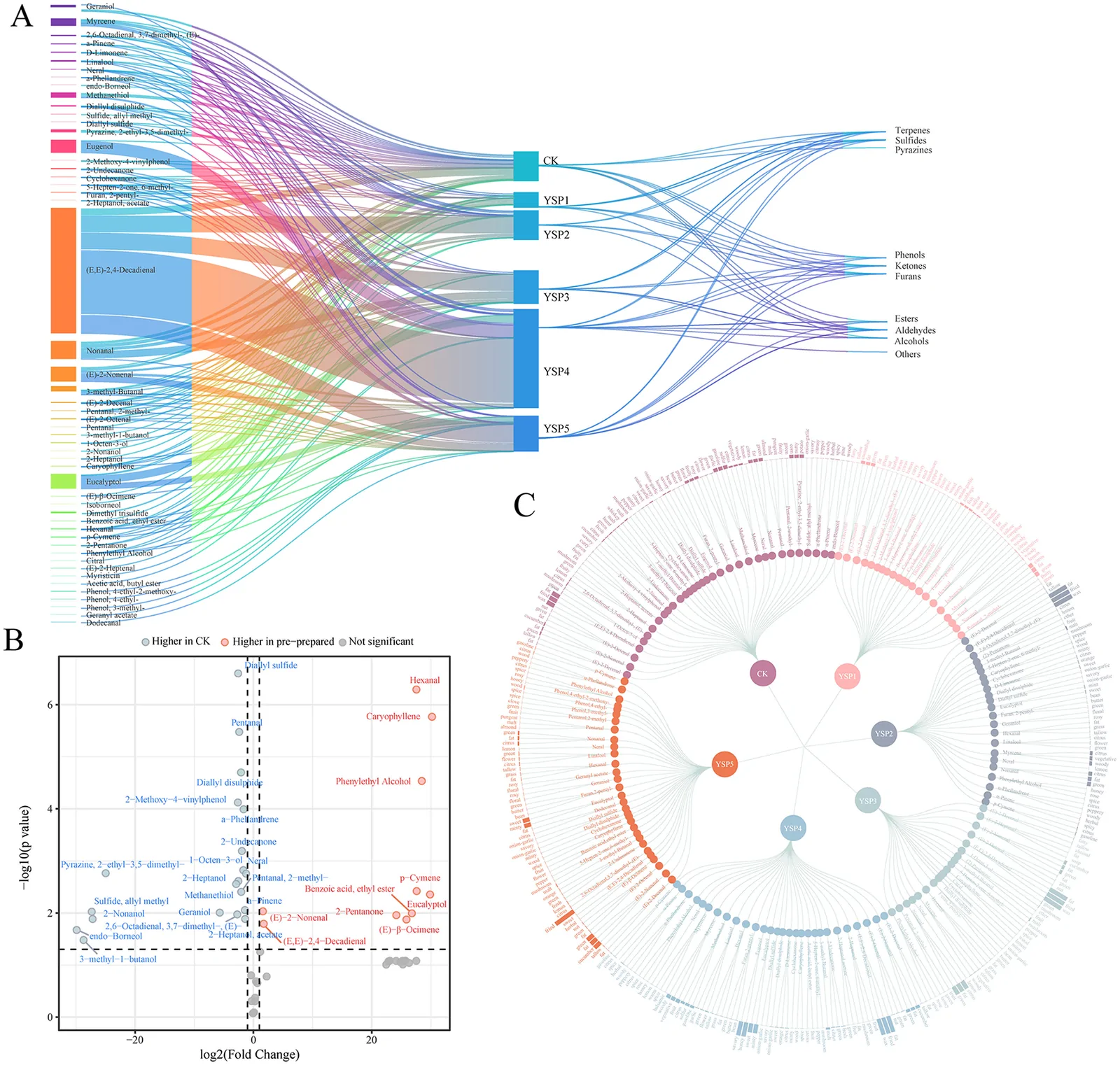
Distribution of OAV-prioritized compounds and volatile flavor differences in freshly stir-fried and commercial ready-to-heat YSP. **(A)** Sankey diagram of compounds with OAV > 1; **(B)** Volcano plots comparing CK and commercial products; **(C)** OAV-derived flavor wheel linking products, candidate odorants, and descriptors.

To further resolve which odorants drove the divergence between CK and the commercial products, volcano plot analysis was performed for the OAV > 1 compounds based on fold change and P values (Figure 5B). Thirty odorants differed significantly, with 20 higher in CK and 10 higher in the commercial-product group. CK-associated compounds included methanethiol, diallyl sulfide, diallyl disulfide, neral, geraniol, and 2-ethyl-3,5-dimethylpyrazine. Their distribution is consistent with stronger Allium, floral-citrus, and roasted notes in CK (41). Hexanal, (E)-2-nonenal, (E,E)-2,4-decadienal, eucalyptol, caryophyllene, phenylethyl alcohol, and ethyl benzoate were higher in the pooled commercial group. These compounds can reflect lipid-derived aldehydes, thermal chemistry, or brand-specific seasoning (42). (E,E)-2,4-decadienal was not unique to commercial products because it also had a high OAV in CK. Higher OAVs in YSP2, YSP3, and YSP5 coincided with hexanal, nonanal, or (E)-2-nonenal. This combination is consistent with stronger lipid-derived aldehyde contributions in those products. The cross-sectional design cannot determine whether the pattern arose from prior oxidation, lipid composition, storage, or reheating-induced release (43). The flavor wheel links products to OAV-prioritized compounds and odor descriptors (Figure 5C). For example, CK was associated with methanethiol (garlic, gasoline, sulfur) and nonanal (fat, green, citrus), whereas the commercial products showed stronger associations with fatty, fried, woody, and seasoning-related descriptors. The wheel is therefore a screening aid rather than a reconstruction of perceived aroma.

### 3.5 Cross-platform associations and association-based interpretation of representative VOCs

The MS-IMS network linked representative VOCs that covaried across products (|ρ| > 0.65, *q* < 0.05; Figure 6A). The network connected terpene, sulfur, alcohol, carbonyl, and furan signals. Several shared VOCs formed a common core, whereas CK-associated terpenes and sulfur compounds were inversely related to product-enriched carbonyl and furan signals. For example, 1-octen-3-ol was inversely associated with 2-furaldehyde and 1-propanol. These correlations describe coordinated product variation and do not establish precursor-product conversion or sensory causation.

**Figure 6:**
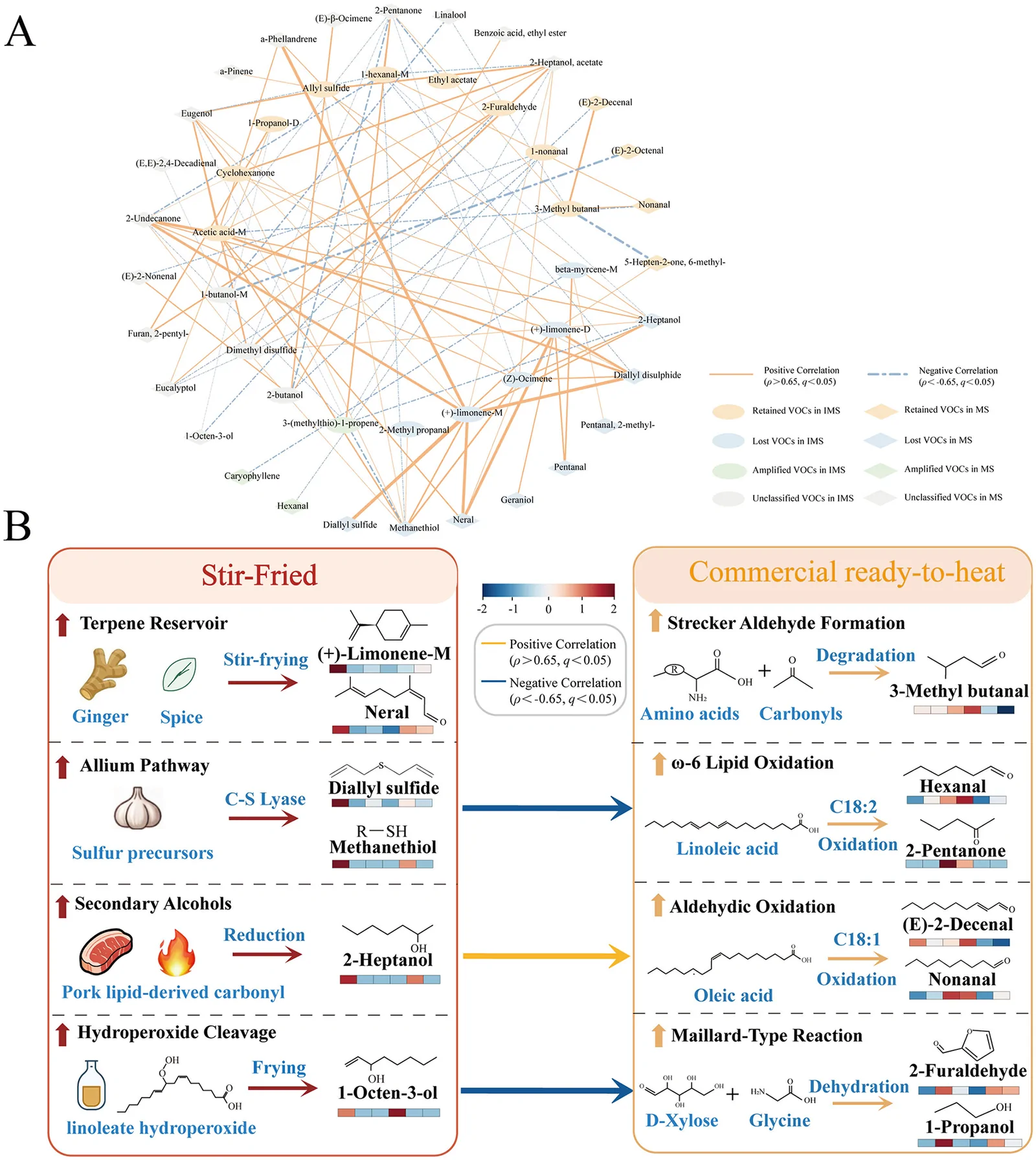
Cross-platform associations among representative VOCs in CK and commercial ready-to-heat YSP products. **(A)** Correlation network of representative headspace solid-phase microextraction GC-MS and HS-GC-IMS signals (|ρ| > 0.65, *q* < 0.05); **(B)** association-based schematic summarizing representative VOC modules associated with CK and commercial ready-to-heat products.

Figure 6B summarizes two association-based chemical modules. The CK-associated module included (+)-limonene and neral, which are commonly associated with spices; methanethiol and diallyl sulfide, which are characteristic Allium-related volatiles; and the lipid-derived alcohols 2-heptanol and 1-octen-3-ol. Their coordinated enrichment in CK was consistent with the rapid release of spice- and Allium-derived volatiles and the presence of short-lived lipid-derived aroma compounds during freshly prepared stir-frying. Their attenuation in most commercial products may therefore contribute to the weaker characteristic and Allium-like aromas observed in the sensory evaluation (44). The commercial-product-associated module included 3-methylbutanal, which is commonly linked to Strecker-type reactions; hexanal and 2-pentanone, which are often associated with linoleic-acid oxidation; nonanal and (E)-2-decenal, which are associated with oleic-acid-related oxidation; and 2-furaldehyde, which can arise from sugar dehydration and Maillard-type reactions (45). Their variable enrichment among YSP1-YSP5 likely reflects combined differences in formulation, processing, storage history, and reheating response. The modules provide an interpretive framework for product benchmarking, not direct evidence of deterioration pathways.

## 4 Conclusion

This study established an exploratory market benchmark for five commercial ready-to-heat YSP products relative to a standardized freshly stir-fried reference. The integrated sensory, FAA, E-nose, E-tongue, HS-GC-IMS, and HS-SPME-GC-MS datasets identified clear product-level variation. Compared with CK, the commercial products were generally characterized by weaker representation of several seasoning-derived terpene and Allium-related signals, together with greater contributions from oxidation-, heat-, and seasoning-associated volatile compounds in selected products. HS-GC-IMS prioritized 25 discriminatory volatile features with VIP > 1, while HS-SPME-GC-MS identified 51 potentially odor-active compounds with OAV > 1 in at least one product. Integration of the HS-GC-IMS and HS-SPME-GC-MS datasets highlighted six potentially odor-active compounds shared across all samples, namely 3-methylbutanal, diallyl disulphide, neral, (E)-2-decenal, 2-pentylfuran, and 6-methyl-5-hepten-2-one. This study has shown that flavor divergence in commercial ready-to-heat YSP is characterized by attenuation of freshly released seasoning-derived top notes, especially terpene- and Allium-derived volatiles, together with increased contributions from oxidation- and heat-associated aldehydes, furans, ketones, pyrazines, and selected sulfur-containing compounds. Although the integrated platform findings provide candidate markers for flavor benchmarking and quality optimization of ready-to-heat traditional dishes, further studies combining controlled processing variables, kinetic validation, and descriptive sensory analysis are needed to clarify how precursor transformation and sensory perception evolve during commercial preparation and reheating. Future studies should identify the remaining unknown volatile markers using high-resolution GC-MS (HR-GC-MS) and comprehensive two-dimensional GC-MS (GC × GC-MS), supported by retention-index matching and authentic-standard confirmation to improve annotation confidence.

## Data Availability

The original contributions presented in the study are included in the article/Supplementary material, further inquiries can be directed to the corresponding authors.
