# Supplementary material for: Flavor fidelity and variation of freshly stir-fried and commercial ready-to-heat Yuxiang shredded pork using integrated sensory and volatile profiling
Source: Front Nutr. 2026 Sep 14;13:1936328. doi: 10.3389/fnut.2026.1936328 (PMC13617055; doi:10.3389/fnut.2026.1936328)
Supplement: Supplementary file 1 [file Supplementary_file_1.docx]

**Flavor fidelity and variation of freshly stir-fried and commercial ready-to-heat Yuxiang shredded pork using integrated sensory and volatile profiling**

**Table S1. Sample designations and commercial states of the freshly stir-fried reference and commercial ready-to-heat YSP products.**

| Sample | Brand | Manufacturer | Commercial format and net content | Principal ingredients recorded from the study package | Storage/handling before analysis |
| --- | --- | --- | --- | --- | --- |
| CK | freshly stir-fried | Sichuan Tourism University laboratory | Freshly prepared; total formulation approximately 497 g | Pork (200 g), celtuce (75 g), hydrated black fungus (20 g), oil (70 g), pickled chili, ginger, garlic, scallion, vinegar, sugar, soy sauce, cooking wine, starch, salt, and monosodium glutamate | Prepared and analyzed on the same day |
| YSP1 | Guyuan | Shijiazhuang Huikang Food Co., Ltd. | Frozen cooking pouch; 200 g | Pork, carrot, bamboo shoot, black fungus, sugar, brewed vinegar, chili, soybean oil, brewed soy sauce, salt, spices, and modified starch/gum additives | Stored frozen. The manufacturer specifies storage below -18 °C and an 18-month frozen shelf life for the 200 g product |
| YSP2 | Fucheng Xiandaojia | Hebei Fucheng Wufeng Food Co., Ltd. | Frozen three-component kit; 300 g (meat 150 g, vegetables 100 g, sauce 50 g) | Study-package record: pork, carrot, black fungus, bamboo shoot, plant oil, soy sauce, sugar, garlic, pickled-chili sauce, vinegar, doubanjiang, ginger, scallion, and related seasonings/additives | Stored frozen according to the product format and study handling; the exact label temperature and shelf life should be checked against the archived study package |
| YSP3 | Weizhixiang | Suzhou Weizhixiang Food Co., Ltd. | Frozen two-component kit; 290 g (main ingredients 250 g, sauce 40 g) | Pork, carrot, bamboo shoot, shiitake mushroom, starch, salt, sugar, chicken seasoning, monosodium glutamate, vinegar, chopped chili, garlic, doubanjiang, scallion, and ginger | Stored frozen according to the product format and study handling; the exact label temperature and shelf life should be checked against the archived study package |
| YSP4 | Shuanghui | Henan Shuanghui Investment & Development Co., Ltd. | Commercial prepared-dish pouch; 223 g in the study package | Pork, water, carrot, bamboo shoot, plant oil, black fungus, sugar, scallion, Pixian doubanjiang, chili oil, chopped chili, yellow rice wine, vinegar, garlic, ginger, and seasonings/additives | Handled as a frozen commercial sample in this study; Stored frozen according to the manufacturer’s instructions |
| YSP5 | Liangnong Xingchu | Anhui Liangnong Food Co., Ltd. | Frozen cooking pouch; 220 g | Bamboo shoot, black fungus, carrot, pork, chili, salt, water, plant oil, Pixian doubanjiang, chicken seasoning, brewed vinegar, sugar, olive oil, and modified starch | Stored frozen according to the product format and study handling; the exact label temperature and shelf life should be checked against the archived study package |

**Table S2. Sensory evaluation standard of YSP.**

| Project | Score | Scoring Criteria |
| --- | --- | --- |
| Appearance | 10 | Uniform color with desirable gloss, and shredded pork is plump in structure; bamboo shoots and black fungus retain their natural color and full structure: 8–10. |
|  |  | Relatively uniform color with acceptable gloss, and shredded pork is slightly wrinkled; bamboo shoots and black fungus retain their natural color but are less plump in structure: 5–7. |
|  |  | Uneven color with moderate gloss, and shredded pork is less plump in structure; bamboo shoots and black fungus are somewhat soft: 3–4. |
|  |  | Dull and dark color, and shredded pork is dry and flattened; bamboo shoots and black fungus are soft and overcooked: 0–2. |
| Characteristic aroma | 10 | Characteristic Yuxiang aroma is intense, typical, and harmonious: 8–10. |
|  |  | Characteristic Yuxiang aroma is obvious and the overall flavor is relatively good: 5–7. |
|  |  | Characteristic Yuxiang aroma is present but weak, with limited intensity or complexity: 3–4. |
|  |  | Characteristic Yuxiang aroma is not obvious, or the overall flavor is poor: 0–2. |
| Allium aroma | 10 | Scallion, ginger, and garlic aroma is intense and well integrated with the overall flavor: 8–10. |
|  |  | Scallion, ginger, and garlic aroma is noticeable and the overall coordination is acceptable: 5–7. |
|  |  | Scallion, ginger, and garlic aroma is weak, or only one note is relatively prominent, with limited coordination: 3–4. |
|  |  | Scallion, ginger, and garlic aroma is not obvious, or is accompanied by unpleasant odor: 0–2. |
| Taste | 10 | Sweet, sour, salty, and pungent tastes are well balanced and harmonious: 8–10. |
|  |  | The overall taste is generally balanced, but one taste is slightly prominent: 5–7. |
|  |  | Taste coordination is weak, and one taste is relatively dominant: 3–4. |
|  |  | Obvious imbalance of taste affects the overall sensory quality: 0–2. |
| Texture | 10 | Pork is tender and palatable, and bamboo shoots and black fungus show their characteristic tenderness and crispness: 8–10. |
|  |  | Pork is slightly tough, and the tenderness and crispness of bamboo shoots and black fungus are less desirable: 5–7. |
|  |  | Pork is slightly tough, and bamboo shoots and black fungus are soft and less crisp: 3–4. |
|  |  | Pork is very tough and lacks meaty flavor, and bamboo shoots and black fungus lack chewiness: 0–2. |

**Table S3. Performance description of the E-nose sensors.**

| **Sensors** | **Performance description** | **Sensors** | **Performance description** |
| --- | --- | --- | --- |
| LY2/LG | Sensitive to oxidizing gas | P40/1 | Sensitive to fluorine |
| LY2/G | Sensitive to ammonia, carbon monoxide | T70/2 | Sensitive to aromatic compounds |
| LY2/AA | Sensitive to ethanol | PA/2 | Sensitive to ethanol, ammonia/organic amines |
| LY2/Gh | Sensitive to ammonia/organic amines | P30/1 | Sensitive to polar compounds (ethanol) |
| LY2/gCT1 | Sensitive to hydrogen sulfide | P40/2 | Sensitive to heteroatom/chloride/aldehydes |
| LY2/gCT | Sensitive to propane/butane | P30/2 | Sensitive to alcohol |
| T30/1 | Sensitive to organic solvents | T40/2 | Sensitive to aldehydes |
| P10/1 | Sensitive to hydrocarbons | T40/1 | Sensitive to chlorinated compounds |
| P10/2 | Sensitive to methane | TA/2 | Sensitive to air quality |

**Table S4. VOC signals putatively identified by HS-GC-IMS in freshly stir-fried and commercial ready-to-heat YSP.**

| Label | CAS | Formula | RT | Peak intensity | | | | | |
| --- | --- | --- | --- | --- | --- | --- | --- | --- | --- |
|  |  |  |  | CK | YSP1 | YSP2 | YSP3 | YSP4 | YSP5 |
| Acids | | | | | | | | | |
| Acetic acid-M | 64-19-7 | C_2_H_4_O_2_ | 1675.13 | 25858.53±432.81^a^ | 23022.03±389.36^bc^ | 20855.91±1060.83^c^ | 17418.86±546.38^d^ | 25347.33±850.80^ab^ | 23899.66±605.44^ab^ |
| Acetic acid-D | 64-19-7 | C_2_H_4_O_2_ | 1670.12 | 7969.07±328.81^ab^ | 8309.88±102.69^ab^ | 6556.73±922.82^b^ | 3880.47±290.14^c^ | 7749.46±737.87^ab^ | 8499.86±333.77^a^ |
| Alcohols | | | | | | | | | |
| 1-Pentanol-M | 71-41-0 | C_5_H_12_O | 907.77 | 1367.39±268.06^c^ | 1559.35±12.22^bc^ | 1494.59±79.93^bc^ | 2210.92±26.09^a^ | 1253.59±33.80^c^ | 1780.30±71.46^ab^ |
| 1-Pentanol-D | 71-41-0 | C_5_H_12_O | 907.77 | 387.18±87.16^abc^ | 326.24±17.57^bc^ | 306.19±9.07^c^ | 439.02±17.29^a^ | 364.96±8.27^abc^ | 380.19±27.42^ab^ |
| 2-Hexanol | 626-93-7 | C_6_H_14_O | 978.22 | 100.54±2.83^c^ | 286.88±18.19^b^ | 76.50±0.99^d^ | 253.99±8.38^b^ | 658.64±38.20^a^ | 755.60±75.13^a^ |
| 1-Butanol, 3-methyl-M | 123-51-3 | C_5_H_12_O | 749.32 | 1792.99±113.28^e^ | 5033.57±45.77^a^ | 2356.27±121.91^d^ | 3260.57±45.26^b^ | 2963.05±65.20^c^ | 3169.58±137.41^bc^ |
| 1-Butanol, 3-methyl-D | 123-51-3 | C_5_H_12_O | 746.79 | 829.13±56.26^d^ | 4646.68±112.15^a^ | 874.13±67.23^d^ | 1384.10±42.99^c^ | 3239.20±51.82^b^ | 1595.09±89.29^c^ |
| 1- butanol-M | 71-36-3 | C_4_H_10_O | 577.75 | 911.34±16.12^d^ | 2777.67±37.03^a^ | 1203.37±70.17^b^ | 882.71±24.86^d^ | 1012.06±16.92^c^ | 573.62±4.38^e^ |
| 1- butanol-D | 71-36-3 | C_4_H_10_O | 578.57 | 225.32±21.85^bcd^ | 1760.38±79.65^a^ | 298.91±34.12^b^ | 227.95±7.18^bc^ | 188.59±9.45^d^ | 191.79±10.55^cd^ |
| 1-Propanol-M | 71-23-8 | C_3_H_8_O | 399.47 | 1520.76±19.09^ab^ | 1611.14±69.28^a^ | 1617.13±16.33^a^ | 1451.40±12.97^bc^ | 1401.99±40.95^c^ | 1646.85±78.06^a^ |
| 1-Propanol-D | 71-23-8 | C_3_H_8_O | 398.38 | 1094.53±67.17^d^ | 3646.49±224.62^a^ | 1147.31±75.11^d^ | 862.32±39.03^e^ | 2722.91±125.07^b^ | 1785.11±102.37^c^ |
| 2- butanol | 78-92-2 | C_4_H_10_O | 380.14 | 1139.15±207.78^c^ | 5586.41±121.57^a^ | 797.82±39.57^d^ | 971.23±28.47^cd^ | 2029.97±80.17^b^ | 5988.04±84.19^a^ |
| 2-Methyl-2-propanol | 75-65-0 | C_4_H_10_O | 300.67 | 1284.94±126.93^a^ | 1330.55±94.08^a^ | 1282.27±49.37^a^ | 1140.67±77.43^ab^ | 1184.16±414.13^ab^ | 615.09±245.99^b^ |
| 1 -hexanol | 111-27-3 | C_6_H_14_O | 1224.34 | 113.04±21.37^c^ | 177.76±11.00^b^ | 138.29±10.97^bc^ | 440.06±6.89^a^ | 157.60±25.28^b^ | 375.43±29.38^a^ |
| (E)-3-hexen-1-ol | 928-97-2 | C_6_H_12_O | 1181.14 | 239.02±26.85^e^ | 374.68±56.43^d^ | 235.11±28.61^e^ | 569.34±66.10^c^ | 771.79±26.71^b^ | 1075.30±29.85^a^ |
| Aldehydes | | | | | | | | | |
| 1-nonanal | 124-19-6 | C_9_H_18_O | 1335.41 | 3533.61±316.83^a^ | 2195.24±167.20^bc^ | 3828.62±352.40^a^ | 2931.44±495.37^ab^ | 1898.28±187.69^c^ | 2050.29±75.45^c^ |
| (E)-2-Heptenal-M | 18829-55-5 | C_7_H_12_O | 1114.46 | 1319.90±128.13^bc^ | 928.88±56.85^de^ | 1125.39±161.69^cd^ | 1543.32±122.47^b^ | 2061.64±43.52^a^ | 895.57±21.85^e^ |
| (E)-2-hexen-1-al | 6728-26-3 | C_6_H_10_O | 781.20 | 746.27±89.30^b^ | 636.18±32.95^bc^ | 1156.85±100.63^a^ | 736.97±53.08^b^ | 654.55±34.73^bc^ | 555.17±41.11^c^ |
| 3-Methyl-2-butenal | 107-86-8 | C_5_H_8_O | 727.83 | 267.10±56.00^d^ | 325.03±14.45^cd^ | 546.81±79.96^a^ | 465.98±18.01^ab^ | 445.26±9.15^abc^ | 354.36±11.24^bcd^ |
| Heptaldehyde-M | 111-71-7 | C_7_H_14_O | 681.45 | 1191.30±189.99^bc^ | 1064.30±9.18^c^ | 1859.08±172.16^a^ | 1478.88±164.36^ab^ | 632.06±49.68^d^ | 948.11±65.63^c^ |
| (Z)-2-Methylpent-2-enal-M | 623-36-9 | C_6_H_10_O | 613.32 | 406.41±32.90^c^ | 1615.26±29.34^a^ | 177.39±9.01^e^ | 232.45±1.39^d^ | 538.41±47.72^b^ | 269.10±11.08^d^ |
| (Z)-2-Methylpent-2-enal-D | 623-36-9 | C_6_H_10_O | 612.49 | 511.88±75.68^b^ | 947.28±72.93^a^ | 246.26±24.08^de^ | 357.12±21.00^c^ | 207.31±18.69^e^ | 325.08±7.87^cd^ |
| (E)-2-Pentenal-M | 1576-87-0 | C_5_H_8_O | 551.63 | 714.24±25.08^d^ | 773.52±4.12^c^ | 864.08±29.31^bc^ | 1452.96±59.73^a^ | 839.30±32.43^c^ | 959.84±52.12^b^ |
| (E)-2-Pentenal-D | 1576-87-0 | C_5_H_8_O | 550.97 | 456.07±81.94^c^ | 376.31±25.22^c^ | 281.01±32.13^d^ | 701.79±73.68^b^ | 1183.87±25.40^a^ | 332.79±12.84^cd^ |
| 1-hexanal-M | 66-25-1 | C_6_H_12_O | 458.59 | 1836.75±359.84^cd^ | 2581.35±42.82^b^ | 3141.28±119.67^ab^ | 3673.42±75.69^a^ | 1673.81±209.39^d^ | 2441.55±331.14^bc^ |
| 1-hexanal-D | 66-25-1 | C_6_H_12_O | 457.25 | 1851.98±1382.20^ab^ | 1871.86±56.19^ab^ | 1940.89±203.49^ab^ | 3512.80±281.59^a^ | 1263.69±151.00^b^ | 1571.04±317.37^ab^ |
| 3-Methyl butanal | 590-86-3 | C_5_H_10_O | 301.21 | 3354.23±150.43^ab^ | 3305.59±69.94^ab^ | 3888.14±127.33^ab^ | 4499.80±221.93^a^ | 2766.58±638.33^b^ | 1446.32±277.80^c^ |
| 2-Methyl propanal | 78-84-2 | C_4_H_8_O | 240.18 | 3373.02±125.09^a^ | 2224.39±78.40^bc^ | 1212.74±133.09^d^ | 1795.86±214.99^c^ | 2416.95±243.55^b^ | 721.01±37.41^e^ |
| Heptaldehyde-D | 111-71-7 | C_7_H_14_O | 673.62 | 252.53±84.14^ab^ | 166.37±18.96^b^ | 372.65±69.25^a^ | 249.57±35.95^ab^ | 114.71±7.97^c^ | 109.53±4.78^c^ |
| 2-methylbutanal | 96-17-3 | C_5_H_10_O | 264.81 | 167.14±7.37^e^ | 778.24±4.04^a^ | 219.23±23.97^d^ | 178.63±8.16^e^ | 583.80±9.00^b^ | 444.17±16.69^c^ |
| (Z)-4-heptenal | 6728-31-0 | C_7_H_12_O | 669.90 | 842.77±130.84^bc^ | 501.42±28.89^d^ | 176.22±19.69^e^ | 793.12±37.92^c^ | 1826.01±114.07^a^ | 1028.14±25.33^b^ |
| (E)-2-Heptenal-D | 18829-55-5 | C_7_H_12_O | 1111.71 | 386.20±13.77^b^ | 277.73±11.40^d^ | 295.72±21.79^cd^ | 351.76±54.42^bc^ | 597.98±32.64^a^ | 287.99±12.52^cd^ |
| Esters | | | | | | | | | |
| Propyl butanoate | 105-66-8 | C_7_H_14_O_2_ | 504.78 | 91.92±29.93^b^ | 257.06±13.61^a^ | 102.05±11.98^b^ | 124.63±7.88^b^ | 201.51±4.14^a^ | 216.28±7.89^a^ |
| Butanoic acid, methyl ester | 623-42-7 | C_5_H_10_O_2_ | 342.84 | 405.43±86.73^cd^ | 523.56±17.42^bc^ | 1467.15±95.16^a^ | 1270.90±99.55^a^ | 322.16±8.58^d^ | 724.66±40.26^b^ |
| Acetic acid propyl ester | 109-60-4 | C_5_H_10_O_2_ | 337.02 | 49.43±5.88^d^ | 413.58±19.33^b^ | 103.03±3.64^c^ | 94.67±15.87^c^ | 46.60±2.18^d^ | 1241.06±20.37^a^ |
| Acetic acid ethyl ester-D | 141-78-6 | C_4_H_8_O_2_ | 284.92 | 9801.06±47.19^b^ | 10132.84±64.86^a^ | 2507.18±97.10^e^ | 5478.74±141.65^d^ | 9975.32±151.13^ab^ | 6815.04±103.39^c^ |
| Acetic acid ethyl ester-M | 141-78-6 | C_4_H_8_O_2_ | 281.24 | 264.51±12.33^d^ | 297.54±7.56^c^ | 374.55±5.25^b^ | 376.93±7.12^b^ | 247.77±7.09^d^ | 491.97±14.93^a^ |
| methyl acetate | 79-20-9 | C_3_H_6_O_2_ | 266.34 | 192.42±11.68^b^ | 304.75±7.43^a^ | 112.97±6.32^d^ | 118.85±7.41^d^ | 162.56±2.49^c^ | 116.42±1.75^d^ |
| Ethyl formate | 109-94-4 | C_3_H_6_O_2_ | 253.74 | 312.58±19.46^bc^ | 493.03±22.36^a^ | 318.98±26.53^bc^ | 296.61±21.58^c^ | 304.09±13.16^bc^ | 350.88±4.40^b^ |
| 3-Methylbutyl 2-methylbutanoate | 27625-35-0 | C_10_H_20_O_2_ | 944.28 | 1962.85±945.56^a^ | 718.27±9.59^b^ | 746.09±19.33^b^ | 829.02±74.05^b^ | 611.86±44.20^b^ | 700.36±36.17^b^ |
| Furans | | | | | | | | | |
| 2-Furaldehyde-M | 98-01-1 | C_5_H_4_O_2_ | 1642.36 | 3141.14±401.07^c^ | 7093.45±184.77^a^ | 608.19±62.78^c^ | 2782.64±82.16^c^ | 6601.39±99.90^a^ | 6234.42±102.28^a^ |
| 2-Furaldehyde-D | 98-01-1 | C_5_H_4_O_2_ | 1640.62 | 342.17±29.32^d^ | 1469.19±126.62^a^ | 1633.70±20.02^b^ | 399.27±43.84^d^ | 1429.80±51.73^a^ | 1000.24±23.83^b^ |
| 2-Ethylfuran | 3208-16-0 | C_6_H_8_O | 343.64 | 1921.01±94.66^a^ | 1695.55±104.64^ab^ | 895.69±84.11^a^ | 1508.64±22.76^c^ | 1719.11±28.04^ab^ | 1506.53±31.06^c^ |
| Tetrahydrofuran-D | 109-99-9 | C_4_H_8_O | 278.41 | 640.05±3.78^b^ | 522.74±18.20^c^ | 694.00±10.94^cd^ | 862.28±4.19^a^ | 643.32±6.03^b^ | 430.94±4.82^d^ |
| Tetrahydrofuran-M | 109-99-9 | C_4_H_8_O | 273.19 | 1023.94±36.99^b^ | 1695.06±40.26^a^ | 1327.00±118.91^a^ | 625.86±8.04^d^ | 997.28±34.74^b^ | 792.27±106.68^c^ |
| 2,5-dimethylfuran | 625-86-5 | C_6_H_8_O | 291.46 | 869.15±54.85^b^ | 461.13±19.43^c^ | 489.10±23.18^cd^ | 1524.72±37.47^a^ | 880.73±180.15^b^ | 1155.61±199.58^ab^ |
| Ketones | | | | | | | | | |
| Cyclohexanone | 108-94-1 | C_6_H_10_O | 1061.27 | 847.24±29.03^b^ | 666.18±70.34^bc^ | 301.91±32.63^ab^ | 397.51±17.67^d^ | 2297.90±210.77^a^ | 570.25±181.27^c^ |
| 1 -hydroxy-2-propanone | 116-09-6 | C_3_H_6_O_2_ | 1061.29 | 209.03±10.47^b^ | 338.74±135.91^ab^ | 1132.98±38.83^c^ | 271.04±30.27^b^ | 583.15±34.08^a^ | 237.61±54.92^b^ |
| 2-Hexanone | 591-78-6 | C_6_H_12_O | 468.93 | 809.24±30.69^d^ | 2439.47±48.47^a^ | 323.25±26.98^e^ | 1653.49±15.55^b^ | 1475.85±93.85^b^ | 1665.37±116.86^b^ |
| 2,3-Pentanedione | 600-14-6 | C_5_H_8_O_2_ | 474.66 | 709.92±57.06^bc^ | 892.12±112.81^b^ | 357.09±17.50^d^ | 490.58±26.69^d^ | 1530.85±135.86^a^ | 555.97±3.87^cd^ |
| 1-Penten-3-one | 1629-58-9 | C_5_H_8_O | 370.62 | 440.34±5.23^c^ | 640.06±51.47^b^ | 4043.18±312.88^ab^ | 265.97±7.67^e^ | 839.74±6.12^a^ | 447.86±11.81^c^ |
| 2-Butanone | 78-93-3 | C_4_H_8_O | 294.53 | 2512.45±362.50^bc^ | 5446.22±98.58^a^ | 6415.62±146.76^a^ | 3432.12±243.58^ab^ | 3033.12±931.35^bc^ | 1659.43±677.81^c^ |
| 2-Butanone, 3-hydroxy-M | 513-86-0 | C_4_H_8_O_2_ | 1012.24 | 2875.32±70.31^d^ | 6140.17±117.97^a^ | 510.81±41.47^bc^ | 5192.04±41.79^b^ | 3382.23±146.64^c^ | 5073.61±144.40^b^ |
| 2-Butanone, 3-hydroxy-D | 513-86-0 | C_4_H_8_O_2_ | 1012.78 | 2156.13±223.04^e^ | 11725.08±181.27^a^ | 271.64±23.06^b^ | 4500.12±131.50^c^ | 3266.18±147.78^d^ | 4724.00±131.27^c^ |
| 2-methyl-2-hepten-6-one | 110-93-0 | C_8_H_14_O | 1158.41 | 1571.60±194.28^a^ | 398.72±48.24^c^ | 485.57±38.36^c^ | 420.65±34.17^c^ | 1232.24±137.06^a^ | 671.93±96.33^b^ |
| 2-Heptanone-M | 110-43-0 | C_7_H_14_O | 664.79 | 1780.44±110.23^a^ | 477.96±64.72^c^ | 199.20±19.93^b^ | 712.88±15.04^b^ | 581.44±10.22^bc^ | 764.35±44.91^b^ |
| 2-Heptanone-D | 110-43-0 | C_7_H_14_O | 662.95 | 1278.65±138.02^a^ | 204.39±4.31^b^ | 8485.23±234.16^b^ | 222.70±25.09^b^ | 193.57±29.36^b^ | 177.60±11.97^b^ |
| Pyrazines | | | | | | | | | |
| 2,6-Dimethyl pyrazine-M | 108-50-9 | C_6_H_8_N_2_ | 1195.15 | 981.17±28.50^d^ | 1606.92±47.43^c^ | 259.80±11.01^e^ | 1070.72±44.21^d^ | 4247.05±46.35^a^ | 1998.90±52.46^b^ |
| 2,6-Dimethyl pyrazine-D | 108-50-9 | C_6_H_8_N_2_ | 1190.28 | 528.30±33.12^d^ | 616.52±37.55^bc^ | 2168.23±107.63^b^ | 564.85±22.62^bcd^ | 1866.41±88.53^a^ | 670.70±51.17^b^ |
| 2-Methylpyrazine | 109-08-0 | C_5_H_6_N_2_ | 945.69 | 369.81±34.27^c^ | 293.62±21.63^de^ | 543.96±11.28^cd^ | 922.16±23.75^a^ | 339.92±6.17^cd^ | 507.84±36.94^b^ |
| 2,3,5,6-Tetramethylpyrazine | 1124-11-4 | C_8_H_12_N_2_ | 1651.05 | 1932.58±211.39^bc^ | 2984.20±40.41^a^ | 1349.19±42.99^d^ | 1697.33±36.39^d^ | 2661.93±21.08^a^ | 1788.63±34.12^cd^ |
| Terpenes | | | | | | | | | |
| (Z)-Ocimene | 3338-55-4 | C_10_H_16_ | 749.95 | 6475.17±699.66^a^ | 2202.25±85.86^c^ | 400.27±33.34^cd^ | 846.32±26.88^e^ | 4404.34±41.40^b^ | 2002.97±118.60^c^ |
| beta-myrcene-M | 123-35-3 | C_10_H_16_ | 613.32 | 1822.22±560.41^a^ | 482.58±86.12^bc^ | 353.20±37.72^b^ | 530.35±11.37^b^ | 384.62±7.58^d^ | 1256.77±49.79^a^ |
| beta-myrcene-D | 123-35-3 | C_10_H_16_ | 613.32 | 824.20±291.65^a^ | 668.03±23.54^a^ | 987.14±107.20^c^ | 722.84±28.22^a^ | 496.71±17.00^ab^ | 621.05±22.81^a^ |
| (+)-limonene-M | 138-86-3 | C_10_H_16_ | 726.95 | 4999.91±357.78^a^ | 625.81±151.09^d^ | 1062.12±53.84^c^ | 578.46±20.03^d^ | 1121.94±43.78^c^ | 1799.51±78.98^b^ |
| (+)-limonene-D | 138-86-3 | C_10_H_16_ | 723.79 | 4078.71±174.35^a^ | 397.94±261.52^d^ | 477.28±26.21^ab^ | 335.26±22.17^d^ | 1771.06±111.88^b^ | 1238.61±36.61^bc^ |
| gamma -Terpinene | 99-85-4 | C_10_H_16_ | 860.92 | 388.34±64.74^b^ | 479.73±153.42^b^ | 1673.61±123.24^b^ | 526.40±26.58^ab^ | 384.45±6.11^b^ | 765.54±11.01^a^ |
| Camphene | 79-92-5 | C_10_H_16_ | 408.64 | 434.58±46.35^c^ | 3859.93±260.92^a^ | 818.20±55.68^c^ | 346.94±33.78^c^ | 1383.31±76.87^b^ | 428.42±14.92^c^ |
| alpha-Pinene | 80-56-8 | C_10_H_16_ | 385.51 | 1135.18±211.54^b^ | 1689.88±66.10^a^ | 256.62±13.85^bc^ | 843.18±6.65^c^ | 1317.58±4.45^ab^ | 1492.86±19.81^a^ |
| beta-Pinene-M | 127-91-3 | C_10_H_16_ | 505.76 | 369.65±91.54^b^ | 253.04±21.85^bc^ | 566.87±22.99^d^ | 204.98±22.34^c^ | 279.22±4.74^b^ | 1889.27±110.32^a^ |
| beta-Pinene-D | 127-91-3 | C_10_H_16_ | 505.06 | 899.45±123.16^bc^ | 649.41±13.86^bcd^ | 274.35±11.91^e^ | 607.67±78.86^cd^ | 1076.69±507.06^ab^ | 1780.15±238.82^a^ |
| beta-myrcene | 123-35-3 | C_10_H_16_ | 545.16 | 742.05±14.52^b^ | 443.97±26.44^c^ | 388.25±27.98^c^ | 309.59±8.90^d^ | 1175.23±23.81^a^ | 412.31±23.00^c^ |
| alpha-terpinolene | 586-62-9 | C_10_H_16_ | 980.13 | 742.89±307.62^b^ | 1036.15±19.30^b^ | 388.25±27.98^c^ | 2585.73±13.54^a^ | 1010.04±68.34^b^ | 3406.00±213.60^a^ |
| Sulfides | | | | | | | | | |
| Allyl sulfide | 592-88-1 | C_6_H_10_S | 582.71 | 4319.12±151.17^b^ | 1900.55±53.72^c^ | 388.90±21.18^e^ | 819.75±89.25^e^ | 5480.02±111.53^a^ | 1350.18±170.94^d^ |
| Dimethyl disulfide | 624-92-0 | C_2_H_6_S_2_ | 496.86 | 3441.93±100.63^b^ | 1097.99±75.79^c^ | 4568.59±19.93^a^ | 249.68±3.01^e^ | 4445.56±137.90^a^ | 780.59±71.07^d^ |
| 3-(methylthio)-1-propene | 10152-76-8 | C_4_H_8_S | 330.52 | 990.85±42.13^d^ | 1905.74±118.61^c^ | 255.63±28.23^c^ | 4302.58±95.30^a^ | 1067.39±5.11^d^ | 2584.46±79.39^b^ |
| Dipropyl disulfide | 629-19-6 | C_6_H_14_S_2_ | 1254.54 | 398.18±27.80^b^ | 340.88±34.63^b^ | 1226.71±87.75^d^ | 252.80±8.10^c^ | 922.11±6.30^a^ | 320.50±35.78^b^ |
| Others | | | | | | | | | |
| styrene | 100-42-5 | C_8_H_8_ | 1019.46 | 403.66±66.99^a^ | 132.11±12.28^cd^ | 10013.14±587.23^b^ | 111.13±16.02^d^ | 239.05±7.25^b^ | 132.13±10.42^cd^ |
| 2- butoxyethanol | 111-76-2 | C_6_H_14_O_2_ | 1371.87 | 10373.13±811.32^b^ | 7167.94±1008.99^c^ | 113.45±1.48^c^ | 8932.90±1657.19^bc^ | 10777.48±842.96^b^ | 40269.69±1593.59^a^ |
| 1,4-Dioxan | 123-91-1 | C_4_H_8_O_2_ | 462.66 | 984.31±191.44^a^ | 267.17±8.05^b^ | 271.64±23.06^b^ | 110.68±8.17^c^ | 993.67±70.67^a^ | 202.79±13.14^b^ |
| 1,8-Cineol | 470-82-6 | C_10_H_18_O | 719.97 | 3145.03±1341.67^a^ | 274.11±12.84^b^ | 271.64±23.06^b^ | 282.67±17.70^b^ | 317.97±34.80^b^ | 326.76±27.03^b^ |

Note: M represents a monomer and D represents a dimer.

Values are means ± standard deviations. Different lowercase letters within a row indicate significant differences among samples (p < 0.05).

**Table S5. VOCs tentatively identified by HS-SPME-GC-MS in freshly stir-fried and commercial ready-to-heat YSP.**

| Compound Name | CAS | molecular formula | SI | RSI | RT | Content (μg/kg) | | | | | |
| --- | --- | --- | --- | --- | --- | --- | --- | --- | --- | --- | --- |
|  |  |  |  |  |  | CK | YSP1 | YSP2 | YSP3 | YSP4 | YSP5 |
| Terpenes | | | | | | | | | | | |
| L-a-Terpineol | 10482-56-1 | C_10_H_18_O | 832 | 937 | 22.63 | 390.11 | 107.23 | ND | ND | 620.07 | ND |
| Geraniol | 106-24-1 | C_10_H_18_O | 832 | 804 | 25.85 | 595.31 | ND | 14.31 | ND | ND | 42.99 |
| Neral | 106-26-3 | C_10_H_16_O | 873 | 982 | 22.33 | 2388.27 | 318.42 | 686.54 | 149.78 | 1966.75 | 1898.39 |
| Caryophyllene oxide | 1139-30-6 | C_15_H_24_O | 726 | 856 | 28.49 | ND | ND | 135.78 | ND | ND | 59.66 |
| Myrcene | 123-35-3 | C_10_H_16_ | 744 | 953 | 9.11 | 454.09 | 110.76 | 788.87 | 101.82 | 1121.36 | ND |
| 2,6-Octadienal, 3,7-dimethyl-, (E)- | 141-27-5 | C_10_H_16_O | 813 | 976 | 23.46 | 5100.49 | 300.21 | 797.17 | ND | ND | 2644.86 |
| Nerolidol | 142-50-7 | C_15_H_26_O | 716 | 805 | 29.66 | 15.94 | ND | ND | ND | 63.16 | ND |
| α-Acorenol | 28400-11-5 | C_15_H_26_O | 939 | 804 | 20.67 | 10.65 | ND | ND | ND | 34.36 | ND |
| (Z)-β-Farnesene | 28973-97-9 | C_15_H_24_ | 926 | 816 | 21.91 | 224.72 | ND | ND | ND | ND | 146.67 |
| γ-Elemene | 29873-99-2 | C_15_H_24_ | 954 | 976 | 25.36 | 98.44 | ND | ND | ND | 56.75 | 53.46 |
| γ-Muurolene | 30021-74-0 | C_15_H_24_ | 959 | 947 | 22.99 | 406.89 | 77.14 | ND | ND | 304.98 | ND |
| (E)-β-Ocimene | 3779-61-1 | C_10_H_16_ | 969 | 950 | 5.66 | ND | 116.64 | ND | ND | ND | 180.47 |
| Eucalyptol | 470-82-6 | C_10_H_18_O | 934 | 801 | 10.24 | ND | 270.19 | 109.65 | ND | 2476.72 | 1888.95 |
| Aromandendrene | 489-39-4 | C_15_H_24_ | 949 | 937 | 21.34 | 37.30 | ND | ND | ND | ND | 49.26 |
| β-Bisabolene | 495-61-4 | C_15_H_24_ | 947 | 974 | 23.21 | ND | 112.73 | 241.08 | 132.27 | ND | 502.62 |
| a-Farnesene | 502-61-4 | C_15_H_24_ | 957 | 803 | 23.73 | 962.75 | ND | 175.98 | ND | ND | 379.52 |
| β-Phellandrene | 555-10-2 | C_10_H_16_ | 951 | 917 | 10.18 | ND | 263.09 | ND | ND | 106.36 | ND |
| Terpinen-4-ol | 562-74-3 | C_10_H_18_O | 928 | 851 | 20.46 | 147.74 | ND | 708.39 | ND | 302.51 | 129.30 |
| D-Limonene | 5989-27-5 | C_10_H_16_ | 958 | 878 | 9.96 | 1491.52 | ND | 2943.77 | ND | 3065.71 | ND |
| Linalool | 78-70-6 | C_10_H_18_O | 899 | 874 | 19.19 | 250.66 | ND | 220.73 | ND | 864.01 | 880.38 |
| Camphene | 79-92-5 | C_10_H_16_ | 856 | 833 | 6.62 | 1989.60 | 311.19 | 603.30 | 322.84 | 4953.97 | 666.84 |
| a-Pinene | 80-56-8 | C_10_H_16_ | 843 | 960 | 5.66 | 1021.76 | ND | 419.81 | ND | 1460.69 | ND |
| Caryophyllene | 87-44-5 | C_15_H_24_ | 854 | 844 | 20.20 | ND | 855.56 | 1790.73 | 577.49 | 1822.00 | 891.37 |
| Isopulegol | 89-79-2 | C_10_H_18_O | 865 | 905 | 19.53 | ND | ND | 38.33 | ND | 49.55 | ND |
| a-Phellandrene | 99-83-2 | C_10_H_16_ | 865 | 809 | 9.02 | 318.34 | ND | 52.22 | ND | 376.01 | 66.68 |
| γ-Terpinene | 99-85-4 | C_10_H_16_ | 884 | 872 | 11.30 | 16.34 | 134.54 | 10.33 | 794.60 | 188.65 | 741.71 |
| p-Cymene | 99-87-6 | C_10_H_14_ | 907 | 872 | 12.07 | ND | ND | 556.18 | ND | 208.65 | 219.18 |
| 3-Carene | 13466-78-9 | C_10_H_16_ | 903 | 813 | 9.43 | 12.76 | ND | ND | ND | ND | ND |
| (E)-a-Bergamotene | 13474-59-4 | C_15_H_24_ | 880 | 966 | 19.33 | 47.37 | ND | ND | ND | ND | ND |
| (Z)-Verbenol | 1845-30-3 | C_10_H_16_O | 842 | 810 | 19.83 | 48.09 | ND | ND | ND | ND | ND |
| Germacrene D | 23986-74-5 | C_15_H_24_ | 963 | 829 | 22.79 | 271.42 | ND | ND | ND | ND | ND |
| (Z)-p-Mentha-2,8-dien-1-ol | 3886-78-0 | C_10_H_16_O | 841 | 916 | 25.55 | 112.67 | ND | ND | ND | ND | ND |
| endo-Borneol | 507-70-0 | C_10_H_18_O | 814 | 975 | 22.67 | 967.75 | ND | ND | ND | ND | ND |
| Isoborneol | 124-76-5 | C_10_H_18_O | 865 | 820 | 22.66 | ND | 89.96 | ND | ND | ND | ND |
| β-Pinene | 127-91-3 | C_10_H_16_ | 937 | 923 | 7.47 | ND | ND | 85.81 | ND | ND | ND |
| Limonene | 138-86-3 | C_10_H_16_ | 810 | 883 | 10.18 | ND | ND | 125.16 | ND | ND | ND |
| Phytol | 150-86-7 | C_20_H_40_O | 936 | 944 | 16.53 | ND | ND | 263.76 | ND | ND | ND |
| Thujone | 546-80-5 | C_10_H_16_O | 898 | 972 | 16.04 | ND | ND | 147.48 | ND | ND | ND |
| o-Cymene | 527-84-4 | C_10_H_14_ | 822 | 904 | 12.05 | ND | ND | ND | 573.32 | ND | ND |
| Citral | 5392-40-5 | C_10_H_16_O | 864 | 812 | 23.46 | ND | ND | ND | 190.74 | ND | ND |
| 2-Carene | 554-61-0 | C_10_H_16_ | 839 | 823 | 12.33 | ND | ND | ND | 78.89 | ND | ND |
| Sulfides | | | | | | | | | | | |
| Trisulfide, di-2-propenyl | 2050-87-5 | C_6_H_10_S_3_ | 886 | 823 | 24.62 | 1251.57 | 725.14 | 1166.86 | 389.02 | 881.33 | 883.16 |
| Diallyl disulphide | 2179-57-9 | C_6_H_10_S_2_ | 886 | 848 | 17.58 | 4980.12 | 331.20 | 1091.24 | 139.59 | 2811.99 | 1320.09 |
| Disulfide, methyl 2-propenyl | 2179-58-0 | C_4_H_8_S_2_ | 872 | 813 | 12.43 | ND | ND | 93.47 | ND | 123.11 | 102.71 |
| Diallyl sulfide | 592-88-1 | C_6_H_10_S | 854 | 839 | 8.72 | 141.21 | ND | 37.06 | ND | 52.89 | 25.24 |
| Disulfide, dipropyl | 629-19-6 | C_6_H_14_S_2_ | 894 | 895 | 14.92 | 193.24 | ND | 78.42 | ND | 128.78 | ND |
| Methanethiol | 74-93-1 | CH_4_S | 937 | 834 | 2.26 | 181.41 | ND | ND | ND | 118.21 | ND |
| Sulfide, allyl methyl | 10152-76-8 | C_4_H_8_S | 823 | 868 | 4.52 | 166.64 | ND | ND | ND | ND | ND |
| Thiirane, methyl- | 1072-43-1 | C_3_H_6_S | 898 | 869 | 3.53 | 2358.72 | ND | ND | ND | ND | ND |
| Trisulfide, methyl 2-propenyl | 34135-85-8 | C_4_H_8_S_3_ | 887 | 834 | 20.21 | 498.27 | ND | ND | ND | ND | ND |
| Desulphosinigrin | 5115-81-1 | C_10_H_17_NO_6_S_2_ | 866 | 974 | 25.01 | 56.06 | ND | ND | ND | ND | ND |
| 1-Allyl-2-isopropyldisulfane | 67421-85-6 | C_6_H_12_S_2_ | 814 | 820 | 16.26 | 210.15 | ND | ND | ND | ND | ND |
| 1-Allyl-3-propyltrisulfane | 33922-73-5 | C_6_H_12_S_3_ | 937 | 898 | 23.31 | ND | 76.29 | ND | ND | ND | ND |
| Dimethyl trisulfide | 3658-80-8 | C_2_H_6_S_3_ | 816 | 950 | 15.01 | ND | 43.05 | ND | ND | ND | ND |
| 2,4-Dithiapentane | 1618-26-4 | C_3_H_8_S_2_ | 826 | 821 | 12.57 | ND | ND | ND | 27.89 | ND | ND |
| 1,2,4,6-Tetrathiepane | 292-45-5 | C_4_H_8_S_4_ | 828 | 925 | 37.53 | ND | ND | ND | 49.32 | ND | ND |
| Propane, 1-(ethynylsulfinyl)- | 121564-27-0 | C_5_H_8_OS | 872 | 927 | 3.53 | ND | ND | ND | ND | ND | 490.26 |
| Octyl thioglycolate | 7664-80-4 | C_10_H_20_O_2_S | 829 | 825 | 6.24 | ND | ND | ND | ND | ND | 65.69 |
| Pyrazines | | | | | | | | | | | |
| Pyrazine, tetramethyl- | 1124-11-4 | C_8_H_12_N_2_ | 846 | 943 | 17.45 | 417.50 | ND | ND | ND | ND | ND |
| Pyrazine, 2-ethyl-3,5-dimethyl- | 13925-07-0 | C_8_H_12_N_2_ | 875 | 931 | 16.70 | 32.59 | ND | ND | ND | ND | ND |
| Pyrazine, 2,3-dimethyl- | 5910-89-4 | C_6_H_8_N_2_ | 880 | 876 | 14.25 | ND | ND | ND | 23.81 | ND | ND |
| Phenols | | | | | | | | | | | |
| 2-Methoxy-4-vinylphenol | 7786-61-0 | C_9_H_10_O_2_ | 831 | 970 | 32.66 | 47.11 | 38.25 | ND | ND | ND | ND |
| Eugenol | 97-53-0 | C_10_H_12_O_2_ | 881 | 825 | 32.09 | 383.85 | ND | ND | ND | 9017.00 | ND |
| Phenol, 2-methoxy-4-(1-propenyl)- | 97-54-1 | C_10_H_12_O_2_ | 915 | 943 | 35.27 | 16.84 | ND | ND | ND | 39.02 | 72.02 |
| Phenol, 4-ethenyl-2,6-dimethoxy- | 28343-22-8 | C_10_H_12_O_3_ | 885 | 908 | 39.24 | 85.88 | ND | ND | ND | ND | ND |
| Phenol, 2-ethyl- | 90-00-6 | C_8_H_10_O | 849 | 988 | 32.23 | ND | ND | ND | 17.81 | ND | ND |
| Methyleugenol | 93-15-2 | C_11_H_14_O_2_ | 831 | 860 | 29.24 | ND | ND | ND | ND | 29.15 | ND |
| Phenol, 3-methyl- | 108-39-4 | C_7_H_8_O | 814 | 805 | 30.55 | ND | ND | ND | ND | ND | 27.21 |
| Phenol, 4-ethyl- | 123-07-9 | C_8_H_10_O | 881 | 894 | 32.23 | ND | ND | ND | ND | ND | 231.58 |
| Phenol, 4-ethyl-2-methoxy- | 2785-89-9 | C_9_H_12_O_2_ | 849 | 848 | 29.54 | ND | ND | ND | ND | ND | 400.19 |
| Others | | | | | | | | | | | |
| Anethole | 104-46-1 | C_10_H_12_O | 869 | 948 | 25.53 | ND | ND | 182.95 | ND | 5163.27 | ND |
| Octadecane, 6-methyl- | 10544-96-4 | C_19_H_40_ | 838 | 811 | 16.82 | ND | ND | 68.47 | ND | 86.25 | ND |
| Tetradecane | 629-59-4 | C_14_H_30_ | 846 | 865 | 15.50 | ND | 247.12 | 51.03 | 62.99 | 387.38 | 666.79 |
| Nonane, 3-methyl- | 5911/4/6 | C_10_H_22_ | 898 | 853 | 4.65 | ND | ND | ND | ND | 45.05 | ND |
| Myristicin | 607-91-0 | C_11_H_14_O_2_ | 874 | 904 | 33.80 | ND | ND | ND | ND | 36.73 | ND |
| Decane | 124-18-5 | C_10_H_22_ | 939 | 839 | 5.26 | ND | ND | ND | ND | ND | 49.22 |
| Ketones | | | | | | | | | | | |
| 2-Pentanone | 107-87-9 | C_5_H_10_O | 832 | 939 | 5.01 | ND | ND | 54.29 | 37.00 | ND | ND |
| 5-Hepten-2-one, 6-methyl- | 110-93-0 | C_8_H_14_O | 873 | 874 | 13.97 | 140.38 | 88.59 | 103.07 | 48.83 | 215.48 | 382.39 |
| Cyclopentanone, 2-methyl- | 1120-72-5 | C_6_H_10_O | 852 | 885 | 12.67 | ND | ND | 950.00 | 950.00 | ND | 950.00 |
| 2-Undecanone | 112-12-9 | C_11_H_22_O | 808 | 839 | 20.40 | 667.74 | ND | ND | ND | 698.91 | 165.12 |
| Acetone | 67-64-1 | C_3_H_6_O | 865 | 908 | 3.73 | 335.21 | 344.50 | 75.52 | 262.24 | 190.12 | 92.38 |
| 3-Heptanone, 5-methyl- | 541-85-5 | C_8_H_16_O | 840 | 850 | 11.72 | ND | ND | ND | 36.54 | ND | ND |
| 2-Propanone, 1-methoxy- | 5878-19-3 | C_4_H_8_O_2_ | 899 | 943 | 4.74 | ND | ND | ND | ND | 11.70 | ND |
| 2-Tridecanone | 593-08-8 | C_13_H_26_O | 889 | 946 | 25.05 | ND | ND | ND | ND | 150.34 | ND |
| 2-Octanone, 1-nitro- | 16067-01-9 | C_8_H_15_NO_3_ | 831 | 932 | 15.75 | ND | ND | ND | ND | ND | 31.04 |
| 2-Hexanone, 6-hydroxy- | 21856-89-3 | C_6_H_12_O_2_ | 857 | 925 | 7.39 | ND | ND | ND | ND | ND | 13.16 |
| Furans | | | | | | | | | | | |
| Furfural | 98-01-1 | C_5_H_4_O_2_ | 915 | 989 | 17.37 | 203.37 | 114.18 | ND | ND | ND | ND |
| Furan, 2-pentyl- | 3777-69-3 | C_9_H_14_O | 850 | 968 | 11.03 | 41.78 | 65.88 | 47.48 | 25.84 | 257.94 | 63.42 |
| 5-Hydroxymethylfurfural | 67-47-0 | C_6_H_6_O_3_ | 818 | 945 | 37.86 | ND | 39.89 | 1446.24 | ND | 94.21 | ND |
| 2-Furanmethanol | 98-00-0 | C_5_H_6_O_2_ | 831 | 879 | 21.91 | ND | ND | ND | 110.82 | ND | ND |
| 3-Furaldehyde | 498-60-2 | C_5_H_4_O_2_ | 820 | 943 | 17.36 | ND | ND | ND | ND | 259.73 | ND |
| Esters | | | | | | | | | | | |
| 2,4-Hexadienoic acid, ethyl ester | 110318-09-7 | C_8_H_12_O_2_ | 842 | 915 | 18.30 | ND | 128.22 | ND | ND | 441.95 | 492.41 |
| Isobornyl acetate | 125-12-2 | C_12_H_20_O_2_ | 800 | 946 | 19.91 | ND | 108.30 | 117.11 | 74.37 | ND | ND |
| 2-Heptanol, acetate | 5921-82-4 | C_9_H_18_O_2_ | 880 | 860 | 11.95 | 93.35 | ND | ND | ND | 178.12 | ND |
| Hexanoic acid, 2-phenylethyl ester | 6290-37-5 | C_14_H_20_O_2_ | 873 | 988 | 25.25 | 65.60 | 26.60 | ND | ND | ND | 80.69 |
| Benzoic acid, ethyl ester | 93-89-0 | C_9_H_10_O_2_ | 858 | 936 | 22.01 | ND | 300.78 | ND | ND | ND | 259.77 |
| Propanoic acid, 2-hydroxy-, ethyl ester | 97-64-3 | C_5_H_10_O_3_ | 878 | 848 | 14.15 | 93.40 | ND | ND | ND | 269.15 | ND |
| 4-Methylpentyl 2-methylbutanoate | 35852-40-5 | C_11_H_22_O_2_ | 855 | 928 | 15.01 | 141.81 | ND | ND | ND | ND | ND |
| 3-Acetoxydodecane | 60826-26-8 | C_14_H_28_O_2_ | 864 | 945 | 9.53 | ND | 10.81 | ND | ND | ND | ND |
| E-2-Hexenyl benzoate | 76841-70-8 | C_13_H_16_O_2_ | 852 | 822 | 21.03 | ND | 39.91 | ND | ND | ND | ND |
| Geranyl isovalerate | 109-20-6 | C_15_H_24_O_2_ | 868 | 823 | 26.85 | ND | ND | 25.95 | ND | ND | ND |
| Hexanoic acid, 2-propenyl ester | 123-68-2 | C_9_H_16_O_2_ | 897 | 820 | 14.83 | ND | ND | ND | 18.37 | ND | ND |
| n-Propyl acetate | 109-60-4 | C_5_H_10_O_2_ | 817 | 912 | 4.89 | ND | ND | ND | ND | 184.53 | ND |
| Acetic acid, butyl ester | 123-86-4 | C_6_H_12_O_2_ | 802 | 896 | 6.97 | ND | ND | ND | ND | 268.53 | ND |
| 2-Nonanol, acetate | 14936-66-4 | C_11_H_22_O_2_ | 866 | 913 | 17.09 | ND | ND | ND | ND | 230.63 | ND |
| Geranyl acetate | 105-87-3 | C_12_H_20_O_2_ | 821 | 805 | 23.94 | ND | ND | ND | ND | ND | 944.54 |
| 3,6-Octadecadiynoic acid, methyl ester | 56554-43-9 | C_19_H_30_O_2_ | 935 | 809 | 12.14 | ND | ND | ND | ND | ND | 82.08 |
| (E)-9-Octadecenoic acid ethyl ester | 6114-18-7 | C_20_H_38_O_2_ | 876 | 935 | 29.82 | ND | ND | ND | ND | ND | 46.63 |
| Hexadecanoic acid, ethyl ester | 628-97-7 | C_18_H_36_O_2_ | 835 | 893 | 33.56 | ND | ND | ND | ND | ND | 12.86 |
| Aldehydes | | | | | | | | | | | |
| Pentanal | 110-62-3 | C_5_H_10_O | 833 | 819 | 2.71 | 39.06 | ND | ND | ND | ND | 36.30 |
| Pentanal, 2-methyl- | 123-15-9 | C_6_H_12_O | 811 | 878 | 3.86 | 58.94 | 26.88 | ND | 35.48 | ND | 7.97 |
| Nonanal | 124-19-6 | C_9_H_18_O | 870 | 820 | 15.38 | 841.42 | 1121.41 | 1015.34 | 2435.34 | ND | 379.64 |
| (E)-2-Nonenal | 18829-56-6 | C_9_H_16_O | 824 | 864 | 18.96 | 52.44 | 136.92 | ND | 123.74 | 137.75 | 372.05 |
| (E,E)-2,4-Decadienal | 25152-84-5 | C_10_H_16_O | 854 | 821 | 25.15 | 331.09 | ND | 795.00 | 800.78 | 3055.97 | 902.48 |
| (E)-2-Octenal | 2548-87-0 | C_8_H_14_O | 803 | 810 | 16.34 | 95.49 | 47.63 | ND | 98.17 | 195.93 | 167.18 |
| (E)-2-Decenal | 3913-81-3 | C_10_H_18_O | 908 | 872 | 21.47 | 817.46 | 419.32 | 701.54 | 1316.49 | 374.51 | 247.51 |
| (Z)-2-Heptenal | 57266-86-1 | C_7_H_12_O | 802 | 825 | 13.63 | 109.12 | ND | ND | ND | 161.22 | ND |
| 3-methyl-Butanal | 590-86-3 | C_5_H_10_O | 878 | 823 | 3.93 | 99.74 | 69.36 | 148.96 | 138.09 | 250.60 | 67.37 |
| Hexanal | 66-25-1 | C_6_H_12_O | 889 | 819 | 7.19 | ND | 138.61 | 98.75 | 161.01 | 277.29 | 275.79 |
| 2-Undecenal | 2463-77-6 | C_11_H_20_O | 849 | 851 | 23.86 | ND | 324.75 | ND | ND | ND | ND |
| (Z)-7-Hexadecenal | 56797-40-1 | C_16_H_30_O | 859 | 921 | 15.17 | ND | 9.50 | ND | ND | ND | ND |
| (E)-Hexadec-2-enal | 22644-96-8 | C_16_H_30_O | 818 | 866 | 16.36 | ND | ND | 53.08 | ND | ND | ND |
| (Z)-13-Octadecenal | 58594-45-9 | C_18_H_34_O | 936 | 884 | 15.69 | ND | ND | 48.87 | ND | ND | ND |
| Benzaldehyde | 100-52-7 | C_7_H_6_O | 830 | 873 | 18.76 | ND | ND | ND | 185.95 | ND | ND |
| (E)-2-Heptenal | 18829-55-5 | C_7_H_12_O | 843 | 812 | 13.64 | ND | ND | ND | 189.18 | ND | ND |
| Dodecanal | 112-54-9 | C_12_H_24_O | 937 | 984 | 18.03 | ND | ND | ND | ND | ND | 147.51 |
| Alcohols | | | | | | | | | | | |
| 2-propyl-1-Heptanol | 10042-59-8 | C_10_H_22_O | 887 | 972 | 12.80 | 56.29 | 25.95 | ND | ND | ND | ND |
| n-Nonadecanol-1 | 1454-84-8 | C_19_H_40_O | 905 | 984 | 15.56 | ND | ND | 76.70 | ND | ND | 66.55 |
| 2-Hexadecanol | 14852-31-4 | C_16_H_34_O | 936 | 822 | 19.06 | 62.00 | 83.30 | ND | ND | 43.94 | ND |
| 1,3-Octanediol | 23433-05-8 | C_8_H_18_O_2_ | 926 | 863 | 7.19 | 91.17 | ND | ND | ND | 71.34 | ND |
| 2-methyl-1-Hexadecanol | 2490-48-4 | C_17_H_36_O | 872 | 929 | 14.66 | ND | 31.51 | 65.95 | ND | 191.25 | 56.89 |
| 1,2,4-Butanetriol | 3068-00-6 | C_4_H_10_O_3_ | 945 | 960 | 7.10 | ND | 10.08 | ND | ND | 99.29 | ND |
| 1-Octen-3-ol | 3391-86-4 | C_8_H_16_O | 937 | 830 | 16.82 | 104.16 | ND | ND | 151.12 | ND | ND |
| 2-ethylhex-2-enol | 50639-00-4 | C_8_H_16_O | 872 | 890 | 16.82 | ND | 49.21 | ND | ND | ND | 116.56 |
| 2-Heptanol | 543-49-7 | C_7_H_16_O | 838 | 925 | 13.46 | 96.09 | ND | ND | ND | 81.22 | ND |
| Phenylethyl Alcohol | 60-12-8 | C_8_H_10_O | 912 | 973 | 27.20 | ND | ND | 252.53 | 502.69 | 468.39 | 570.03 |
| 3-Decyn-2-ol | 69668-93-5 | C_10_H_18_O | 934 | 841 | 19.41 | ND | 95.65 | ND | 155.45 | ND | ND |
| 1-Octyn-3-ol | 818-72-4 | C_8_H_14_O | 830 | 951 | 10.52 | ND | ND | ND | ND | 115.34 | 112.00 |
| 3-methyl-1-butanol | 123-51-3 | C_5_H_12_O | 809 | 895 | 10.50 | 429.85 | ND | ND | ND | ND | ND |
| 2-Nonanol | 628-99-9 | C_9_H_20_O | 925 | 844 | 18.50 | 149.47 | ND | ND | ND | ND | ND |
| 1,3-Propanediol, 2-ethyl-2-(hydroxymethyl)- | 77-99-6 | C_8_H_18_O_3_ | 888 | 973 | 12.14 | 16.17 | ND | ND | ND | ND | ND |
| 2-methyl-1-Propanol | 78-83-1 | C_4_H_10_O | 891 | 982 | 7.66 | 46.60 | ND | ND | ND | ND | ND |
| 2-Hexyl-1-octanol | 19780-79-1 | C_14_H_30_O | 801 | 805 | 16.54 | ND | 103.18 | ND | ND | ND | ND |
| 3-Octanol, 6-ethyl- | 19781-27-2 | C_10_H_22_O | 835 | 877 | 19.17 | ND | ND | ND | 332.80 | ND | ND |
| Cyclodecanol | 1502-05-2 | C_10_H_20_O | 813 | 811 | 18.02 | ND | ND | ND | ND | 159.16 | ND |
| 1-Penten-3-ol, 4-methyl- | 4798-45-2 | C_6_H_12_O | 946 | 930 | 12.12 | ND | ND | ND | ND | 68.84 | ND |
| 1-Pentanol, 4-amino- | 927-55-9 | C_5_H_13_NO | 868 | 849 | 2.56 | ND | ND | ND | ND | 102.60 | ND |
| 1-Pentanol, 2-ethyl-4-methyl- | 106-67-2 | C_8_H_18_O | 849 | 923 | 5.38 | ND | ND | ND | ND | ND | 31.28 |
| 2-Undecanol | 1653-30-1 | C_11_H_24_O | 830 | 954 | 13.46 | ND | ND | ND | ND | ND | 23.35 |
| Acids | | | | | | | | | | | |
| Sorbic Acid | 110-44-1 | C_6_H_8_O_2_ | 808 | 804 | 31.84 | ND | 519.48 | ND | ND | 2037.80 | 183.17 |
| Acetic acid | 64-19-7 | C_2_H_4_O_2_ | 879 | 982 | 17.08 | 437.72 | 525.27 | ND | ND | ND | ND |
| Nonanoic acid | 112-05-0 | C_9_H_18_O_2_ | 884 | 856 | 32.06 | ND | 160.66 | ND | ND | ND | ND |
| 9-Hexadecenoic acid | 2091-29-4 | C_16_H_30_O_2_ | 869 | 953 | 26.22 | ND | ND | 32.39 | ND | ND | ND |
| Cyperanic acid | 117869-89-3 | C_15_H_22_O_2_ | 837 | 976 | 33.04 | ND | ND | ND | 34.33 | ND | ND |
| 2-Methylthioacetic acid | 2444-37-3 | C_3_H_6_O_2_S | 876 | 805 | 26.05 | ND | ND | ND | 543.66 | ND | ND |
| (Z)-Vaccenic acid | 506-17-2 | C_18_H_34_O_2_ | 873 | 804 | 26.13 | ND | ND | ND | ND | 33.37 | ND |
| Propanedioic acid, 3-thienyl- | 21080-92-2 | C_7_H_6_O_4_S | 821 | 816 | 40.12 | ND | ND | ND | ND | ND | 43.11 |
| Benzoic acid | 65-85-0 | C_7_H_6_O_2_ | 871 | 976 | 37.05 | ND | ND | ND | ND | ND | 200.46 |

Note: RT, retention time; SI, Similarity Index; RSI, Reverse Search Index; ND, not detected. GC-MS identities are tentative library-based annotations. All nondetected sample–compound combinations are labeled ND.

**Table S6. Odor activity values of compounds detected by HS-SPME-GC-MS.**

| Compound Name | Flavor description | Odor Threshold (ug/kg) | CK | YSP1 | YSP2 | YSP3 | YSP4 | YSP5 |
| --- | --- | --- | --- | --- | --- | --- | --- | --- |
| (E,E)-2,4-Decadienal | fried, wax, fat | 0.16 | 2069.29 | ＜1 | 4968.75 | 5004.89 | 3474.84 | 5640.52 |
| (E)-2-Nonenal | cucumber, fat, green | 0.19 | 276.01 | 720.61 | ＜1 | 651.26 | 725.00 | 1958.14 |
| Eucalyptol | mint, sweet | 1.10 | ＜1 | 245.63 | 99.69 | ＜1 | 2251.56 | 1717.22 |
| Nonanal | fat, citrus, green | 1.10 | 764.93 | 1019.47 | 923.04 | 2213.95 | ＜1 | 345.13 |
| Linalool | citrus, flower, green | 6.00 | 41.78 | ＜1 | 36.79 | ＜1 | 144.00 | 146.73 |
| 3-methyl-Butanal | malt | 0.50 | 199.48 | 138.72 | 297.92 | 276.19 | 501.20 | 134.74 |
| Phenol, 4-ethyl-2-methoxy- | woody | 4.40 | ＜1 | ＜1 | ＜1 | ＜1 | ＜1 | 90.95 |
| 2,6-Octadienal, 3,7-dimethyl-, (E)- | citrus lemon | 32.00 | 159.39 | 9.38 | 24.91 | ＜1 | ＜1 | 82.65 |
| (E)-2-Octenal | green, nut, fat | 3.00 | 31.83 | 15.88 | ＜1 | 32.72 | 65.31 | 55.73 |
| Hexanal | grass, tallow, fat | 5.00 | ＜1 | 27.72 | 19.75 | 32.20 | 55.46 | 55.16 |
| Diallyl disulphide | onion-garlic | 30.00 | 166.00 | 11.04 | 36.37 | 4.65 | 93.73 | 44.00 |
| p-Cymene | gasoline, citrus | 5.00 | ＜1 | ＜1 | 111.24 | ＜1 | 41.73 | 43.84 |
| Geraniol | floral, rosy，fruity | 1.00 | 595.31 | ＜1 | 14.31 | ＜1 | ＜1 | 42.99 |
| 2-Undecanone | orange, fresh, green | 5.00 | 133.55 | ＜1 | ＜1 | ＜1 | 139.78 | 33.02 |
| Neral | lemon | 100.00 | 23.88 | 3.18 | 6.87 | 1.50 | 19.67 | 18.98 |
| Dodecanal | fat, citrus | 10.00 | ＜1 | ＜1 | ＜1 | ＜1 | ＜1 | 14.75 |
| (E)-2-Decenal | fatty,tallow | 17.00 | 48.09 | 24.67 | 41.27 | 77.44 | 22.03 | 14.56 |
| Caryophyllene | wood, spice | 64.00 | ＜1 | 13.37 | 27.98 | 9.02 | 28.47 | 13.93 |
| Phenol, 4-ethyl- | spice | 21.00 | ＜1 | ＜1 | ＜1 | ＜1 | ＜1 | 11.03 |
| Furan, 2-pentyl- | green，bean, butter | 5.80 | 7.20 | 11.36 | 8.19 | 4.45 | 44.47 | 10.94 |
| Geranyl acetate | floral, rosy | 150.00 | ＜1 | ＜1 | ＜1 | ＜1 | ＜1 | 6.30 |
| 5-Hepten-2-one, 6-methyl- | pepper, mushroom | 68.00 | 2.06 | 1.30 | 1.52 | 0.72 | 3.17 | 5.62 |
| (E)-β-Ocimene | sweet, herb | 34.00 | ＜1 | 3.43 | ＜1 | ＜1 | ＜1 | 5.31 |
| Pentanal, 2-methyl- | green，fruity | 1.60 | 36.83 | 16.80 | ＜1 | 22.18 | ＜1 | 4.98 |
| Benzoic acid, ethyl ester | flower, fruit | 53.00 | ＜1 | 5.68 | ＜1 | ＜1 | ＜1 | 4.90 |
| Pentanal | almond, malt, pungent | 12.00 | 3.25 | ＜1 | ＜1 | ＜1 | ＜1 | 3.03 |
| Phenol, 3-methyl- | spice, clove | 15.00 | ＜1 | ＜1 | ＜1 | ＜1 | ＜1 | 1.81 |
| a-Phellandrene | citrus, woody, peppery | 40.00 | 7.96 | ＜1 | 1.31 | ＜1 | 9.40 | 1.67 |
| Phenylethyl Alcohol | honey, spice, rose | 560.00 | ＜1 | ＜1 | 0.45 | 0.90 | 0.84 | 1.02 |
| Diallyl sulfide | Savory，onion-garlic | 100.00 | 1.41 | ＜1 | 0.37 | ＜1 | 0.53 | 0.25 |
| Eugenol | savory， honey | 2.50 | 153.54 | ＜1 | ＜1 | ＜1 | 3606.80 | ＜1 |
| Myrcene | woody, vegetative, citrus | 1.20 | 378.41 | 92.30 | 657.40 | 84.85 | 934.47 | ＜1 |
| Methanethiol | sulfur, gasoline, garlic | 0.20 | 907.05 | ＜1 | ＜1 | ＜1 | 591.07 | ＜1 |
| a-Pinene | herbal, spicy | 14.00 | 72.98 | ＜1 | 29.99 | ＜1 | 104.33 | ＜1 |
| D-Limonene | sweet, orange, citrus | 34.00 | 43.87 | ＜1 | 86.58 | ＜1 | 90.17 | ＜1 |
| Acetic acid, butyl ester | sweet,fruity | 58.00 | ＜1 | ＜1 | ＜1 | ＜1 | 4.63 | ＜1 |
| 2-Heptanol, acetate | fatty，green，fruity | 87.00 | 1.07 | ＜1 | ＜1 | ＜1 | 2.05 | ＜1 |
| 2-Heptanol | fruity，green，mushroom | 41.00 | 2.34 | ＜1 | ＜1 | ＜1 | 1.98 | ＜1 |
| Myristicin | spice, warm, balsamic | 25.00 | ＜1 | ＜1 | ＜1 | ＜1 | 1.47 | ＜1 |
| 1-Octen-3-ol | green，mushroom | 1.50 | 69.44 | ＜1 | ＜1 | 100.75 | ＜1 | ＜1 |
| Citral | lemon | 40.00 | ＜1 | ＜1 | ＜1 | 4.77 | ＜1 | ＜1 |
| (E)-2-Heptenal | soap, fat, almond | 40.00 | ＜1 | ＜1 | ＜1 | 4.73 | ＜1 | ＜1 |
| 2-Pentanone | ether, fruit | 10.00 | ＜1 | ＜1 | 5.43 | 3.70 | ＜1 | ＜1 |
| Dimethyl trisulfide | sulfur, fish, cabbage | 0.10 | ＜1 | 430.51 | ＜1 | ＜1 | ＜1 | ＜1 |
| 2-Methoxy-4-vinylphenol | savory，curry | 3.00 | 15.70 | 12.75 | ＜1 | ＜1 | ＜1 | ＜1 |
| Isoborneol | woody，minty，camphor | 8.50 | ＜1 | 10.58 | ＜1 | ＜1 | ＜1 | ＜1 |
| Pyrazine, 2-ethyl-3,5-dimethyl- | nutty,corn,potato | 0.04 | 814.85 | ＜1 | ＜1 | ＜1 | ＜1 | ＜1 |
| 3-methyl-1-butanol | whiskey, malt, burnt | 4.00 | 107.46 | ＜1 | ＜1 | ＜1 | ＜1 | ＜1 |
| Sulfide, allyl methyl | savory，onion-garlic | 22.00 | 7.57 | ＜1 | ＜1 | ＜1 | ＜1 | ＜1 |
| endo-Borneol | pine woody | 180.00 | 5.38 | ＜1 | ＜1 | ＜1 | ＜1 | ＜1 |
| 2-Nonanol | cucumber | 58.00 | 2.58 | ＜1 | ＜1 | ＜1 | ＜1 | ＜1 |

Note: Odor descriptions and thresholds were obtained from http://www.odour.org.uk. Odor descriptions and odor thresholds in water were obtained from the cited odor database. OAVs were calculated using semi-quantitative VOC concentrations and are interpreted as approximate indicators of relative odor potency. Values <1 indicate calculated OAVs below one and should not be interpreted as nondetection.
